# A synthetic NKG2A engager enables long-term persistence of HLA-deficient allogeneic engineered Tregs

**DOI:** 10.1038/s44321-026-00507-4

**Published:** 2026-08-27

**Authors:** Tingxi Guo, Kaya R Epstein, Maegan E Hoover, Nathan W Zammit, Jennifer Y Yam, Scott Hussell, Sophia Hernandez, Abigail Doherty, Payam Zarin, Martina Sassone-Corsi, Lindsay Webb, Gene I Uenishi, Thomas Wickham, Christopher L Moore

**Affiliations:** GentiBio Inc, Cambridge, MA 02140 USA

**Keywords:** Immunology, Stem Cells & Regenerative Medicine

## Abstract

Allogeneic cell therapies offer a scalable and off-the-shelf alternative to the autologous approach, but immune rejection, particularly by natural killer (NK) cells following human leukocyte antigen (HLA) ablation, remains a major barrier to their persistence. Here, we report an improved synthetic NKG2A engager to selectively inhibit NKG2A⁺ NK cells while avoiding activation of NKG2C⁺ subsets, thereby overcoming a key limitation of the natural ligand HLA-E. Engineered regulatory T cells (EngTregs) lacking HLA and expressing the engager were protected from in vitro NK cell-mediated cytotoxicity more effectively than previously reported NK inhibitory strategies. In humanized mouse models, EngTregs persisted for up to 12 weeks, whereas unprotected cells were rapidly rejected. Incorporation of the engager into a clinically compatible dual-AAV EngTregs preserved Treg identity and function while conferring resistance to immune rejection. Together, these findings establish the improved NKG2A engager as an effective synthetic immune-evasion strategy and provide a clinically translatable approach to enable durable persistence of off-the-shelf EngTreg therapies.

## Synopsis

A synthetic NKG2A engager overcomes natural killer (NK) cell-mediated rejection of HLA knockout (KO) allogeneic cells and enables long-term persistence of engineered regulatory T cells (EngTregs) in vitro and in vivo, thereby advancing off-the-shelf cell therapies.

- A synthetic NKG2A engager was engineered to selectively inhibit NKG2A⁺ NK cells, overcoming the functional limitation of HLA-E in activating NKG2C⁺ NK cells.
- Robust protection of HLA KO EngTregs from NK-mediated rejection was demonstrated using the NKG2A engager, providing superior protection over HLA-E and CD47.
- Multi-months persistence of allogeneic HLA KO EngTregs with the engager was achieved in CD34+ hematopoietic stem cell humanized mice, whereas unmodified or HLA KO only cells were rapidly rejected.
- Immune evasion using HLA KO with NKG2A engager was maintained in a clinically relevant EngTreg designed for type 1 diabetes without compromising phenotype or suppressive function.

## Abstract (web summary)

A synthetic NKG2A engager overcomes natural killer (NK) cell-mediated rejection of HLA knockout (KO) allogeneic cells and enables long-term persistence of engineered regulatory T cells (EngTregs) in vitro and in vivo, thereby advancing off-the-shelf cell therapies.

## Graphical abstract

**Figure:**
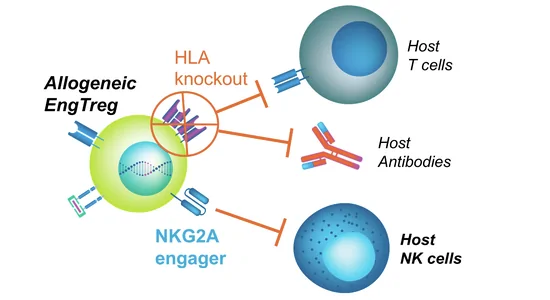

The paper explained **Problem**

Allogeneic “off‑the‑shelf” engineered regulatory T cell (EngTreg) therapies could transform the treatment of autoimmune and inflammatory diseases, but their clinical utility is limited by immune rejection, particularly by natural killer (NK) cells when human leukocyte antigen (HLA) molecules are removed to avoid allorejection by the adaptive immune system.

**Results**

Here, a synthetic engager molecule targeting NKG2A was developed to robustly inhibit NK cell-mediated rejection. EngTregs lacking HLA and expressing this engager showed markedly improved resistance to NK‑mediated killing and achieved sustained persistence for up to 12 weeks in humanized mouse models, whereas unprotected allogeneic cells were rapidly rejected. Incorporation into a clinically relevant EngTreg product preserved regulatory function and phenotype.

**Impact**

These findings establish an immune evasion strategy that could enable durable persistence of allogeneic EngTreg therapies, advancing their feasibility as scalable, accessible treatments for autoimmune diseases. This engineering strategy may also be applicable to other allogeneic cell therapies to address broader indications.

## Introduction

Ex vivo engineered cell therapies have demonstrated clinical success in treating refractory malignant and autoimmune diseases (Chung et al, 2024; Patel et al, 2025). Emerging cell therapy platforms such as engineered regulatory T cells (EngTregs) can treat a wide range of autoimmune and inflammatory diseases (Honaker et al, 2020; Tewari et al, 2024; Tripathi et al, 2025; Uenishi et al, 2024; Yang et al, 2022). A clinical example of the EngTreg platform is GNTI-122 for treating type 1 diabetes (NCT06919354), which is produced by modifying autologous bulk CD4 T cells with two knock-ins at the *FOXP3* and *TRAC* loci using site-specific nucleases and DNA template delivered via adeno-associated virus (AAV). These cells demonstrate a stable Treg phenotype facilitated by constitutively and highly expressed endogenous FOXP3, along with synthetic cytokine support and specificity for the pancreatic islet autoantigen IGRP (Uenishi et al, 2024). While autologous cell therapies are capable of achieving durable efficacy, they could suffer from delayed time to treatment, high cost, and subpar starting material quality for generating optimal therapeutic products (Torikai and Cooper, 2016). Off-the-shelf allogeneic cell therapies derived from healthy donors with scaled-up production can overcome the limitations of conventional autologous therapies. A critical challenge in the allogeneic therapy field is the host rejection of the allogeneic product, which must be addressed for durable and effective therapies (Martin et al, 2024). This barrier similarly applies to ex vivo Treg modalities. Mitigating immune rejection of allogeneic products will unlock the full potential of off-the-shelf cell therapies, including EngTregs, with greater quality and accessibility compared to autologous products. Importantly, scaled-up allogeneic cell therapy can greatly reduce the cost-of-goods per dose compared to autologous (Harrison et al, 2019), to a range competitive with conventional biologics while having the potential for a superior therapeutic index.

Several methods have been employed to mitigate rejection of allogeneic cells, which is heavily mediated by alloreactive T cells and antibody responses targeting mismatched human leukocyte antigens (HLAs) (Ingulli, 2010; Wood and Goto, 2012). Historically, immune suppressive agents have been used to mitigate rejection of allografts, but present toxicities associated with non-selective systemic immune suppression (Richard Ruiz, 2015). Matching, or partial matching, of HLA alleles between the donor and recipient can also prolong allograft survival (Williams et al, 2016). In a skin graft rejection model, allogeneic Tregs with partial HLA matching to PBMC responders provided longer graft protection than allogeneic Tregs with complete mismatch (McCallion et al, 2025). However, HLA matching presents logistical complexity, necessitating a bank of multiple donor materials (Gragert et al, 2014), and is still susceptible to rejection through the presentation of minor histocompatibility antigens even if HLAs are perfectly matched (Goulmy, 1996). Molecular engineering of the therapeutic cell product to confer cell-intrinsic protection to host immunity can avoid the biological and practical drawbacks of systemic immune suppression and HLA matching.

Given the importance of HLAs as key target antigens in rejection, the first step for effective immune evasion engineering is eliminating HLA class I and class II expression on allogeneic cells. Genetic knockout (KO) of critical genes such as *B2M* for HLA class I and *CIITA* for HLA class II with readily available gene engineering tools enables effective evasion of HLA-targeted rejection mechanisms, but the loss of HLA also promotes rapid natural killer (NK) cell-mediated rejection (Kagoya et al, 2020). Robust NK inhibition combined with HLA KO can theoretically generate allogeneic cells capable of evading host immune rejection and achieving long-term durability.

Among immune evasion molecules used to reduce rejection of HLA KO allogeneic cells, two natural inhibitory ligands have advanced to the clinical stage of development: HLA-E (Degagne et al, 2024; Gornalusse et al, 2017; Jo et al, 2022; McCallion et al, 2025) and CD47 (Carlsson et al, 2025; Hu et al, 2024). CD47 binds to the inhibitory receptor SIRPα and is a well-established potent inhibitor of myeloid cells (Logtenberg et al, 2020). Preclinical data indicate CD47 overexpression prevents killing by NK cells (Deuse et al, 2021; Deuse et al, 2019), although its effectiveness as an NK inhibitor has not been consistent across different models (Hiura et al, 2025; Pizzato et al, 2024; Wang et al, 2021; Zhang et al, 2025). On the other hand, HLA-E is a prominent NK receptor ligand with consistent biological effects and can be effective in the appropriate setting. For example, allogeneic HLA KO Tregs with ectopic HLA-E expression demonstrated graft protection on par with autologous Tregs and were significantly better than unmodified allogeneic Tregs (McCallion et al, 2025). Nevertheless, HLA-E binds to both the inhibitory receptor NKG2A and the activating receptor NKG2C expressed on NK cells (Braud et al, 1998), which poses a problem as an immune evasion tool; the protective benefit of HLA-E can be outweighed by its detrimental stimulatory effect, depending on the NKG2A/NKG2C composition of the host NK compartment (Jo et al, 2022; Zhang et al, 2025).

In addition to natural ligands, recent engineering efforts have produced synthetic proteins that elicit inhibitory signals in human NK cells (Gravina et al, 2023; Hiura et al, 2025; Zhang et al, 2025). Here, we describe a synthetic NKG2A engager that selectively delivers inhibitory signals to NK cells and enables superior persistence of HLA KO allogeneic EngTregs compared to field-standard NK inhibitors. We deployed this immune evasive engineering strategy on the GNTI-122 architecture to develop an allogeneic EngTreg product GNTI-148, where the HLA KO and NKG2A engager would mitigate HLA-targeted T cell and antibody alloresponses and NK-mediated missing-self responses, respectively. This technology will catalyze the clinical utility of allogeneic EngTreg therapies for autoimmune and inflammatory diseases, and potentially other allogeneic cell therapies across a variety of indications, by enabling the persistence required for durable efficacy.

## Results

### HLA-E inconsistently protects HLA class I-deficient T cells and can exacerbate rejection

Mitigation of NK cell-mediated missing-self rejection is critical in enabling persistence of HLA KO T cell therapies, including EngTregs. To determine whether the magnitude of missing-self rejection response is associated with a particular NK subset, peripheral blood mononuclear cells (PBMCs) from 12 donors were procured and co-cultured with B2M KO EngTregs to measure killing capacity over one day (Fig. 1A). PBMCs were also phenotyped for the frequencies of various immune subsets. Killing of B2M KO target cells did not significantly relate to the frequency of bulk B cells, monocytes, T cells, or NK cells within PBMCs (Fig. 1B and EV1). When a similar analysis was performed with specific subsets of NK cells known to contribute to missing-self rejection (NKG2A+, inhibitory KIR+, and ILT2+) (Anfossi et al, 2006; Yawata et al, 2008), a significant positive relationship was only observed between the extent of B2M KO target killing and frequency of NKG2A+ NK cells (Fig. 1C), implicating these cells as potential drivers of missing-self responses. No significant relations between killing and frequencies of inhibitory KIR+ or ILT2+ NK cells were observed (Fig. EV1F). These observations support other reports showing NKG2A+ NK cells respond more strongly than NKG2A- cells when stimulated with HLA-deficient target T cells (Hammer et al, 2024; Perica et al, 2025).

**Figure 1:**
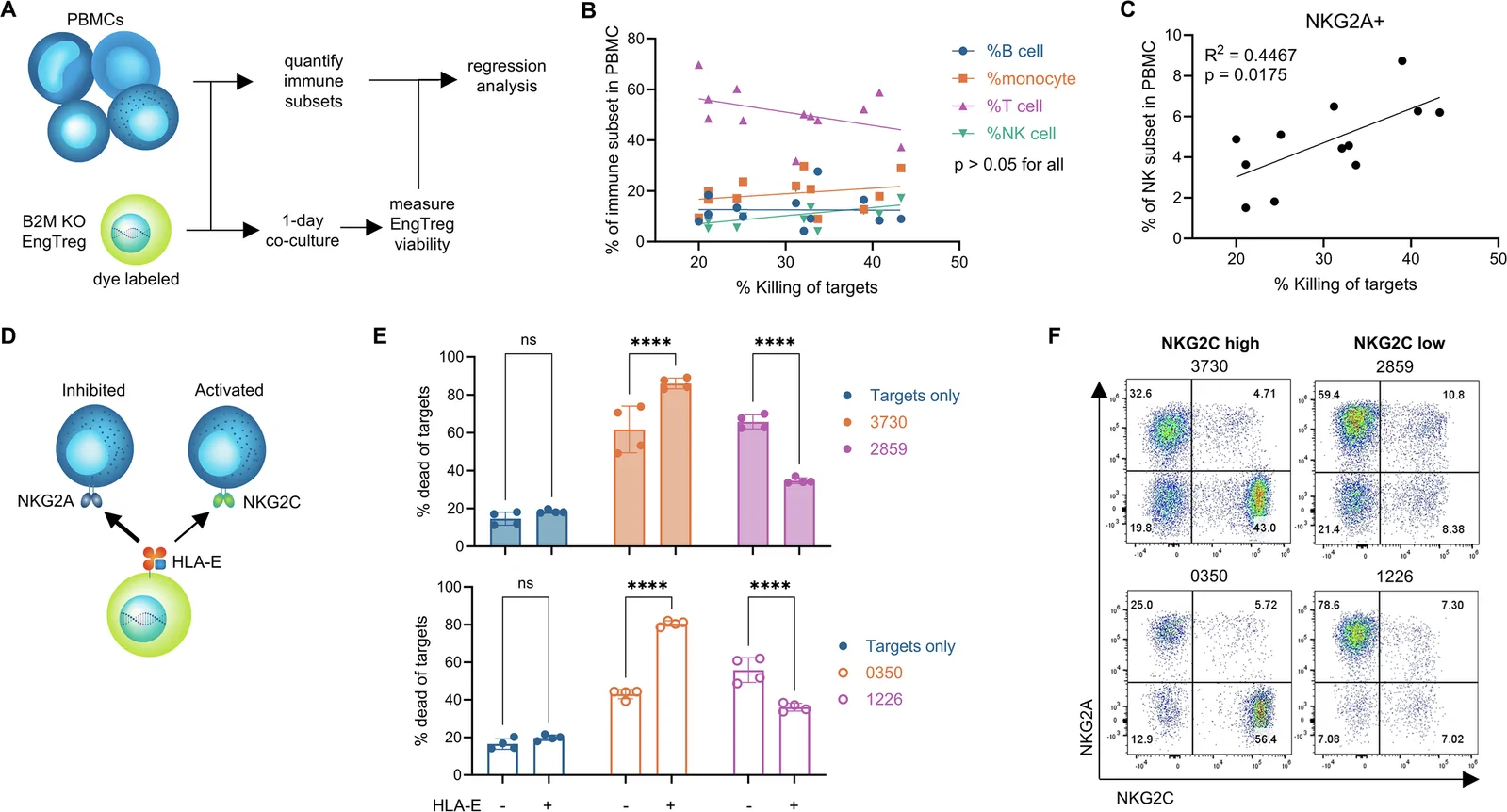
Frequency of NKG2A+ NK cells associated with killing of B2M KO EngTregs. (**A**) Schematic depicting assay system with PBMC responders and B2M KO EngTreg targets. (**B**) Frequencies of B cells (CD19+), monocytes (CD14+), T cells (CD3+), or NK cells (lineage-CD56+) in PBMCs from 12 donors were plotted against the percentage of B2M KO target cells killed by the PBMC in 1 day. (**C**) The frequency of NKG2A + NK cells in PBMC was plotted against the percent of B2M KO target cells killed by the PBMC. Plots in panels (**B**, **C**) were analyzed by simple linear regression (*n* = 12 unique PBMC donors). (**D**) Schematic depicting the mechanism of HLA-E for inhibiting and stimulating NK cells. (**E**) Cell death of B2M KO EngTreg, untransduced or expressing HLA-E via lentivirus, were quantified after co-culture with indicated PBMC. Target only cultures quantified baseline cell death. Error bars denote mean ± SD of two independent experiments performed in technical duplicates (*n* = 4). ns *p* > 0.05, ****p < 0.0001 by two-way ANOVA with Bonferroni’s multiple comparisons test. (**F**) NKG2A and NKG2C phenotype gated on live NK cells (lineage-CD56+) in the four donors used in panel (**E**). Source data are available online for this figure.

**Figure EV1:**
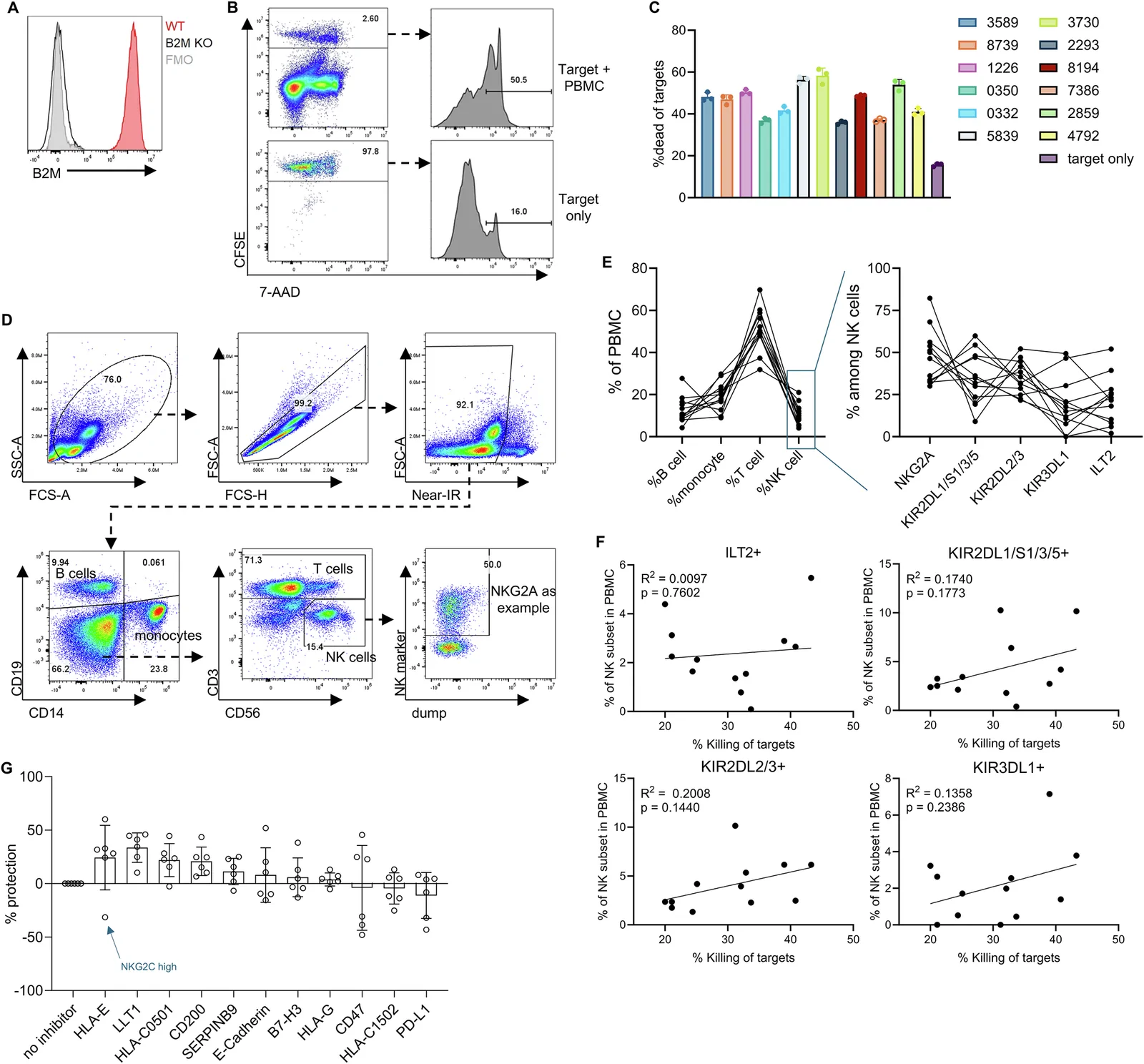
B2M KO target cell killing and associations with various immune subsets. (**A**) Surface B2M phenotype of B2M KO EngTregs used in Fig. 1. (**B**) Representative flow plots used for quantifying cell death in CFSE-labeled B2M KO targets with or without PBMCs. Gated on singlets. (**C**) Cell death was quantified as shown in panel (**B**). Error bars denote mean ± SD of technical triplicates. (**D**) Representative gating strategy for quantifying immune subsets in PBMCs. (**E**) Frequencies of indicated immune subsets were quantified in the PBMCs from the 12 donors tested in panel (**C**). Each line denotes one unique donor. (**F**) Linear regression analyses of the percent killing of B2M KO EngTreg targets with the frequency of the indicated NK subset. (**G**) B2M KO Jurkat cells were lentivirally transduced with the indicated transgene and co-cultured with NK cells from six different donors. Percent protection was quantified as described in Methods. Error bars denote mean ± SD.

Given that inhibiting NKG2A+ NK cells is critical for mitigating missing-self rejection responses, we tested the prototypical NKG2A ligand, a single-chain HLA-E with HLA-G leader peptide and B2M covalently linked to the HLA-E heavy chain(Gornalusse et al, 2017)—herein referred to as HLA-E. In addition to its inhibitory role, HLA-E also engages the activating NKG2C receptor (Fig. 1D). Consistent with past reports (Hiura et al, 2025; Jo et al, 2022; Perica et al, 2025), HLA-E reduced killing of B2M KO targets when the responders had low frequency of NKG2C+ NK cells, but exacerbated killing if the responder donor possessed high levels of NKG2C+ NK cells (Fig. 1E,F). In a side-by-side comparison, other naturally occurring NK inhibitors were not more potent for NK inhibition than HLA-E (Fig. EV1G).

### Synthetic NKG2A-specific engager provides consistent and superior protection over HLA-E

Recognizing the limitation of HLA-E as an NK inhibitory ligand and the critical need to inhibit NKG2A+ NK cells, we developed synthetic NK ligands using membrane-anchored NKG2A binders. We assembled five disclosed NKG2A selective clones (U.S. Patent Pub. No. 2011/0052606A1, US Patent No. 8,206,709, PCT Pub. No. WO 2023/114176A2, and US Patent No. 11,274,150), generated single-chain fragment variable (scFv) domains in both the heavy-light (HL) and light-heavy (LH) orientations with CD8A hinge and PDGFRA transmembrane domains (Gravina et al, 2023), and expressed them on B2M KO EngTregs via lentivirus with an LNGFR marker (Fig. 2A). All constructs were efficiently expressed on the target cells and NKG2A engagers demonstrated binding to the recombinant cognate molecule (Fig. EV2A). Protection of target cells from PBMC killing were measured in a 1-day co-culture assay relative to untransduced B2M KO cells. With the exception of clone NKG2A.9-based constructs, all NKG2A engagers consistently protected B2M KO targets across ten different PBMC responders compared to HLA-E, which did not protect target cells from killing by three of the PBMC donors (Figs. 2B and EV2B). Based on the consistency of the protective effect (Fig. 2B) and the clinical precedence of monalizumab (van Hall et al, 2019), we selected Mona-HL as the lead NKG2A engager for subsequent experiments.

**Figure 2:**
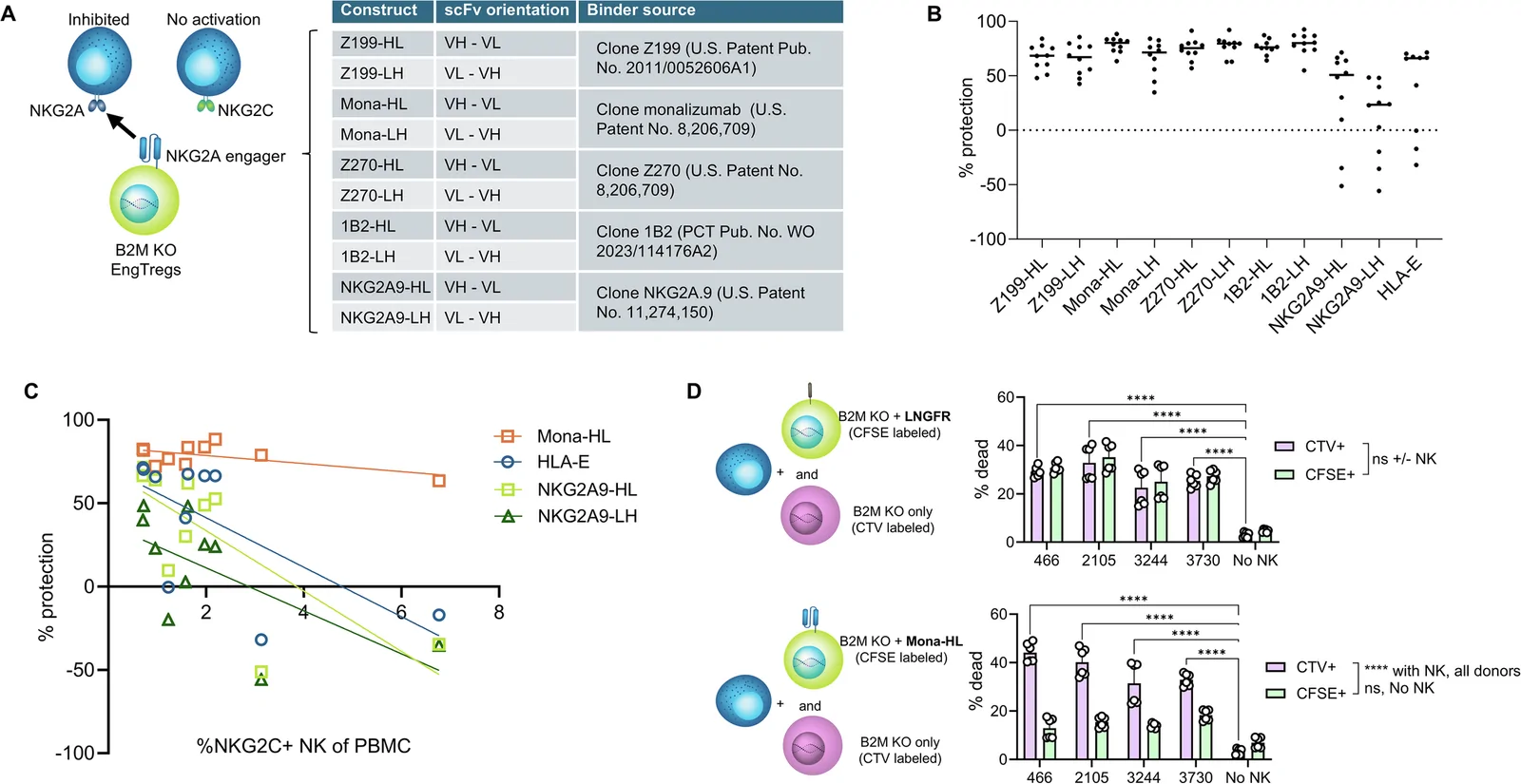
Synthetic NKG2A engager consistently protected B2M KO EngTreg. (**A**) Schematic depicting the mechanism of NKG2A engager and a summary of single-chain fragment variable-based binders used to generate NKG2A engagers. (**B**) Each NKG2A engager candidate was cloned into a lentiviral vector, transduced to B2M KO EngTregs, and target cells were co-cultured with PBMCs from ten donors as described in Fig. 1A for 1 day. Percent protection was calculated by normalizing killing of transduced cells to untransduced control after subtracting baseline cell death (see Methods). Each point represents the average of technical duplicates for each PBMC donor (*n* = 10 unique PBMC donors). Bars denote the median. (**C**) Percent protection across the ten PBMC donors tested were plotted against the frequency of NKG2C+ NK cells for the indicated test articles. (**D**) Schematics depicting assay conditions are shown on the left. CFSE-labeled B2M KO targets transduced with a control transgene (top) or Mona-HL NKG2A engager (bottom) were co-cultured with CTV-labeled bystander B2M KO EngTregs and purified NK cells from four donors for 18 h, at a ratio of 1:1:10 of CFSE+ target:CTV+ target:NK. The percent of dead cells were quantified by flow cytometry among each target cell population. Error bars denote mean ± SD of two independent experiments performed in technical triplicates (*n* = 6). ns *p* > 0.05, ****p < 0.0001 by two-way ANOVA with Bonferroni’s multiple comparisons test. Source data are available online for this figure.

**Figure EV2:**
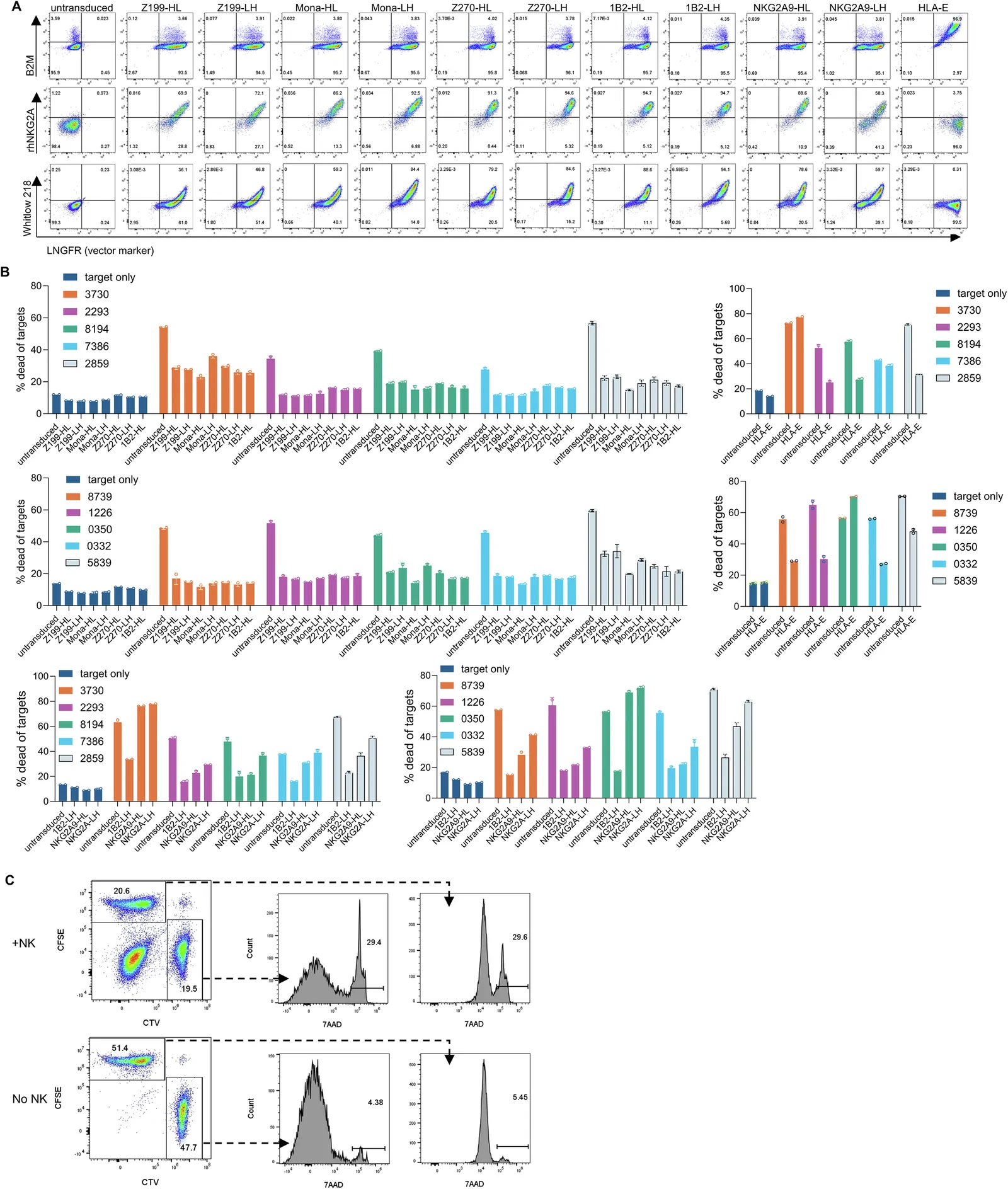
Expression and protective eff ect of NKG2A engagers. (**A**) Phenotype of untransduced control and transduced targets, gated on live cells. All lentiviral vectors were marked with truncated LNGFR. NKG2A engagers were detected using recombinant human NKG2A (rhNKG2A) and anti-Whitlow 218 linker antibody. Note that HLA-E is not expected to bind soluble recombinant NKG2A, given the low physiological binding affinity. (**B**) Cell death was quantified and used to calculate percent protection for each condition shown in Fig. 2B. Error bars denote mean ± SD of technical duplicates. Samples assayed on different plates are shown in the respective bar graphs. (**C**) Representative gating strategy for a control LNGFR-transduced EngTreg target used to quantify cell death in Fig. 2D.

Notably, the protective effect of Mona-HL was consistent across all PBMC responders with varying NKG2C+ NK cell frequency, while an inverse trend was observed for the protective effect of HLA-E (Fig. 2C). Although protective in some donors, the NKG2A.9-based engagers behaved similarly to HLA-E (Fig. 2C). To investigate the cell-intrinsic protective effect of Mona-HL, a three-way co-culture was established using purified NK cells and two B2M KO EngTreg targets, each labeled with a respective dye (CFSE or CTV). CFSE-labeled targets expressed either a control molecule (LNGFR alone) or Mona-HL, and CTV-labeled EngTregs served as untransduced bystander target cells (Figs. 2D and EV2C). After an overnight co-culture, NK cells effectively killed bystander B2M KO CTV+ cells in the presence of Mona-HL expressing targets (Fig. 2D), demonstrating that NKG2A-engager minimally interferes with NK function against bystander targets. Again, Mona-HL protected target cells consistently across the tested donors (Fig. 2D). These results demonstrate that a synthetic NKG2A engager can overcome the biological limitation of HLA-E, while retaining cell-intrinsic protective effects.

### Improved NKG2A engager enables superior persistence of HLA KO allogeneic EngTregs in a robust in vitro rejection system

To stringently evaluate the robustness of immune evasion technologies, we sought to develop an in vitro co-culture assay that captures strong allogeneic rejection responses by T and NK cells. For developing such an assay, we used LNGFR-marked HLA+ or HLA KO (B2M and CIITA KO) EngTregs and co-cultured them with autologous or allogeneic PBMCs at two different ratios (Fig. 3A,B). The presence of LNGFR+ target cells was tracked longitudinally in culture up to nine days. Based on the expected kinetics and mechanism of T cell-mediated rejection, HLA+ EngTregs were able to expand initially from day 0 to 3, but were significantly reduced by day 7 in the presence of allogeneic PBMCs. HLA+ targets persisted throughout the duration of the assay with autologous PBMCs, consistent with the absence of rejection in autologous systems (Fig. 3B, orange lines). In contrast, HLA KO targets were unable to expand, as expected with rapid NK-mediated rejection, and were significantly reduced by day 7 in the presence of both autologous and allogeneic responders (Fig. 3B, black lines). These results demonstrate that this PBMC co-culture system robustly captures both T and NK-mediated rejection effects, and readouts at day 0 (baseline) and day 7 (endpoint) sufficiently measure these effects. Similar week-long co-culture assays have been employed in other studies to measure allogeneic rejection responses (Mo et al, 2021; Wang et al, 2021).

**Figure 3:**
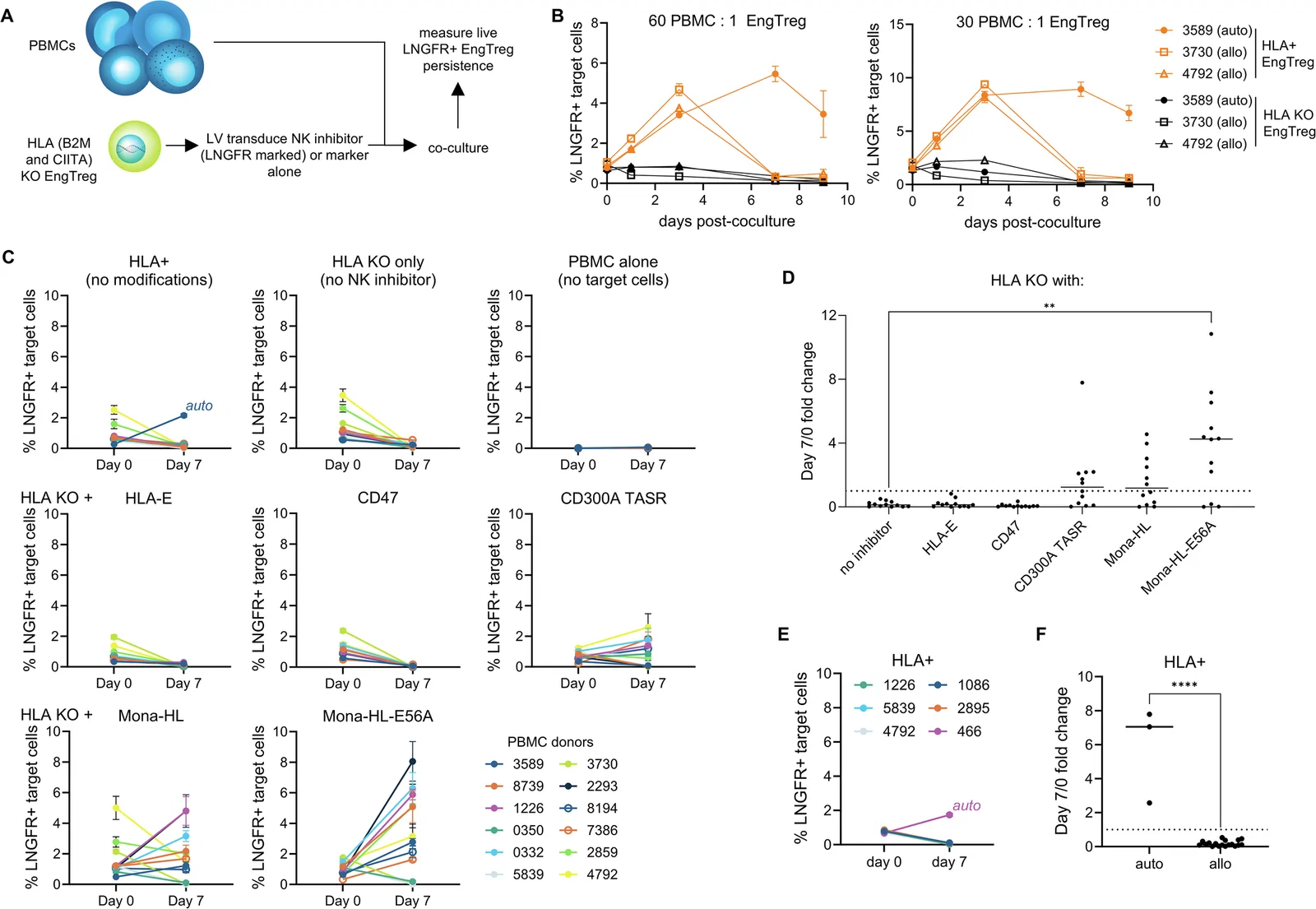
Improved NKG2A engager (Mona-HL-E56A) enabled superior persistence of allogeneic EngTregs compared to competing NK inhibitors in a robust in vitro rejection assay. (**A**) Schematic depicting the assay system. (**B**) Assay was developed using LNGFR-marked HLA+ (orange lines) or HLA KO (black lines) EngTreg targets co-cultured with autologous (auto) or two allogeneic (allo) PBMCs at two PBMC:EngTreg ratios. The frequency of live LNGFR+ targets were quantified in replicate plates on day 0, 1, 3, 7, and 9 after co-culture. (**C**) EngTregs retaining HLA (HLA+) or with HLA KO only were transduced with LNGFR marker (top row). HLA KO EngTregs were also transduced with lentivirus encoding an NK inhibitor with the LNGFR marker (middle and bottom rows). EngTreg target cells were co-cultured with PBMCs from 12 donors, one of which was autologous (3589), at a ratio of 40 PBMC:1 EngTreg. The frequency of LNGFR+ target cells were measured at day 0 and 7 among live CD14- cells by flow cytometry. PBMCs were cultured alone for baseline measurement of LNGFR signals. (**D**) Fold enrichment based on % LNGFR+ cells was calculated for each HLA KO EngTreg test article shown in (**C**). Each point represents the change in mean from day 0 to 7 for one of the tested PBMCs in panel (**C**) (*n* = 12 unique PBMC donors). A dashed line is shown at y = 1, and bars denote the median. **p < 0.01 by one-way ANOVA paired for each PBMC responder with Dunn’s multiple comparisons test. No significant differences compared to the no inhibitor control were observed for any other test article. (**E**) Biological replicate of the experiment described in panel (**C**) using an independent batch of HLA+ EngTreg target cells tested against 6 PBMC responders. (**F**) Persistence of HLA+ EngTreg was quantified, as described in panel (**D**), across three independent experiments from panels (**B**) (60:1), (**C**, **E**). *n* = 3 (2 unique responder-target donor pairs, one of which was an independent replicate) for autologous, and *n* = 18 (16 unique responder-target donor pairs, two of which were independent replicates) for allogeneic co-cultures. ****p < 0.0001 by unpaired *t*-test. Error bars denote mean ± SD of technical duplicates or triplicates in all panels. Source data are available online for this figure.

Using this developed assay, we tested the protective effect of various NK inhibitors with HLA KO (Figs. 3C and EV3A). EngTregs with HLA KO only or unmodified HLA were included as biological controls. Target cells were co-cultured with the autologous PBMC (donor 3589) or 11 allogeneic PBMCs, and the frequency of LNGFR+ cells were measured over time. EngTreg target cells were generated from donor 3589 with the HLA genotype encoding C1, C2, and Bw4 alleles involved in human NK cell licensing (Yawata et al, 2008). NK cells were present for the duration of the experiment in culture (Fig. EV3B). Again, we observed the expected complete or near-complete rejection of HLA+ targets by allogeneic responders and HLA KO only targets by all responders (Fig. 3C). In this system, HLA-E and CD47 did not offer substantial protection against any of the donors (Fig. 3C), suggestive of rigorous rejection effects in this 7-day system versus shorter co-cultures where HLA-E did facilitate some protection (Fig. 2B). The Mona-HL NKG2A engager and a recently reported CD300A TASR (Zhang et al, 2025) both enabled persistence at noticeably higher levels than HLA-E and CD47 (Fig. 3C,D). In addition to the parental Mona-HL design, we tested a version of Mona-HL with an E56A point mutation in the scFv heavy chain, which reportedly reduces affinity of the antibody (U.S. Patent No. 8,206,709) and reduced the binding strength to recombinant NKG2A as a membrane-anchored engager (Fig. EV3C). Reducing the affinity of Mona-HL was motivated by the precedents of excessively high receptor binding affinities negatively impacting productive signaling(Irving et al, 2012; Mao et al, 2022). HLA KO EngTregs expressing the Mona-HL-E56A engager was the only test article that persisted at significantly higher levels versus HLA KO only targets across the donors tested (Figs. 3C,D and EV3D). A biological replicate using HLA+ target cells from an independent donor also showed persistence only in the autologous setting (Fig. 3E), demonstrating the consistency of the assay system for investigating allogeneic rejection (Fig. 3F). Collectively, we identified a robust in vitro allogeneic rejection model and used it to identify an improved NKG2A engager (Mona-HL-E56A) that enabled superior persistence of allogeneic EngTregs compared to some of the other reported NK inhibitors.

**Figure EV3:**
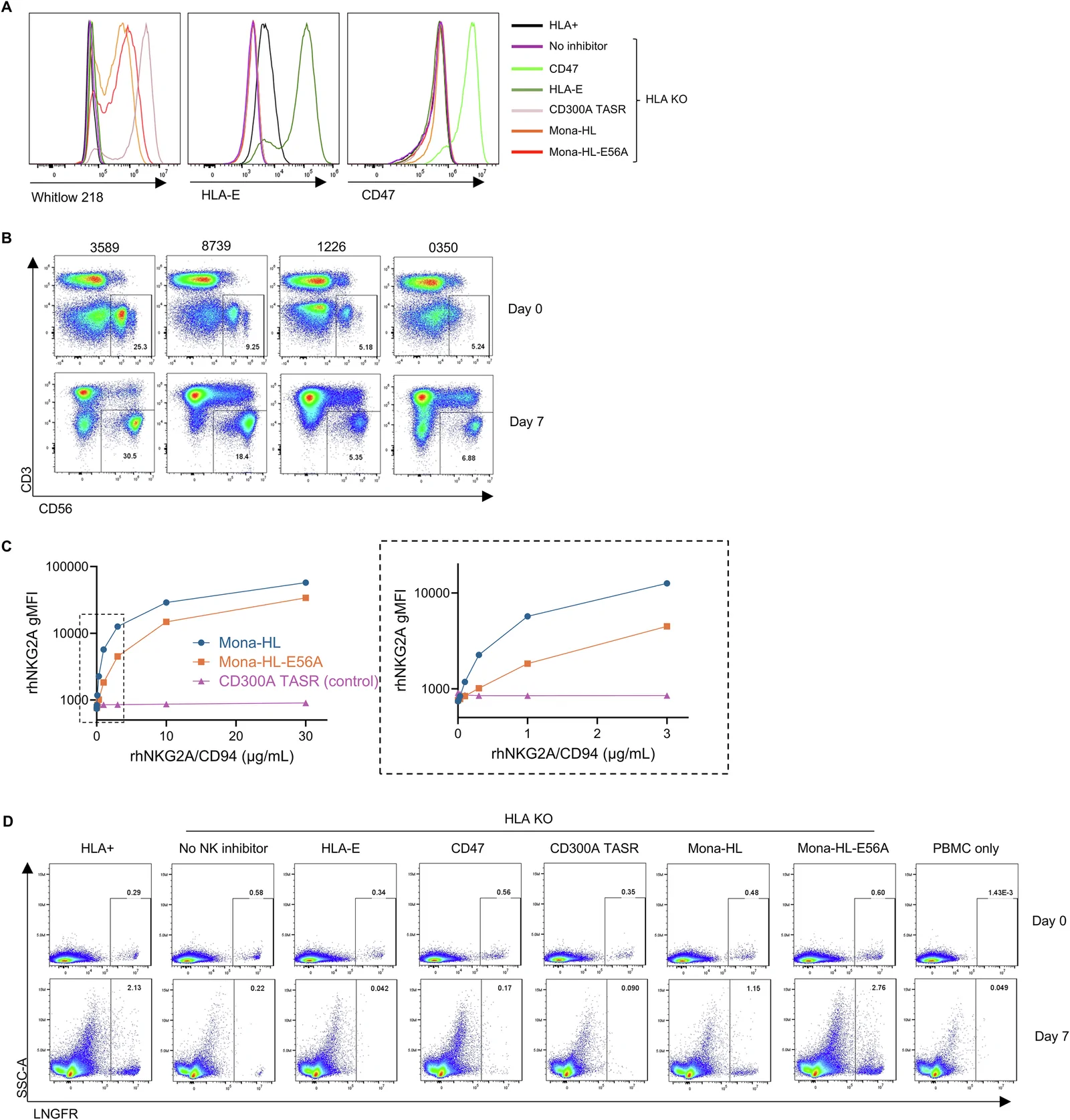
Expression and function of Mona-HL-E56A engager versus other NK inhibitors. (**A**) Surface expression of the indicated NK inhibitor on EngTreg test articles used in Fig. 3C. (**B**) Representative flow plots for four PBMC donors co-cultured with HLA KO only target cells demonstrating the presence of CD3-CD56 + NK cells among live, CD14-, LNGFR- cells over the course of the experiment in Fig. 3C. (**C**) Cognate ligand binding strength of Mona-HL and Mona-HL-E56A NKG2A engagers were measured using a cell-based binding assay using titrated concentrations of His-tagged recombinant human NKG2A(rhNKG2A)/CD94, followed by fluorescently labeled anti-His antibody. Fluorescence among LNGFR+ transduced cells were quantified. Cells expressing CD300A TASR were used as a negative control. Error bars denote mean ± SD of technical duplicates or triplicates. The dashed box on the right shows an inset of the low concentration range from the graph on the left. (**D**) Representative flow plots gated on total live CD14- cells for quantifying the percent of LNGFR+ EngTregs shown in Fig. 3C using the PBMC autologous to the EngTregs.

### Improved NKG2A engager enables long-term persistence of allogeneic EngTregs in humanized mice

Having demonstrated robust NK inhibitory effect of the NKG2A engager in vitro, we sought to investigate its function in protecting HLA KO EngTregs in vivo. First, PBMCs were co-infused with allogeneic HLA KO EngTregs with or without the Mona-HL-E56A engager (Appendix Fig. S1A), or infused alone as a negative control to nod-scid gamma (NSG) with transgenic human IL-15 (NSG-IL15) to support human NK function in vivo (Abeynaike et al, 2023; Aryee et al, 2022). Presence of allogeneic EngTregs were measured on day 0 and day 14 post-infusion (Fig. 4A). Total human CD45+ cells were comparable in the peripheral blood across groups and timepoints (Fig. 4B), indicating no engraftment biases between the groups. HLA KO EngTregs can be distinguished from the allogeneic PBMCs by surface B2M expression. Based on frequency and counts, HLA KO EngTregs expressing the engager persisted at higher levels by day 14 compared to HLA KO only EngTregs, despite starting at comparable levels on day 0 (Fig. 4C,D).

**Figure 4:**
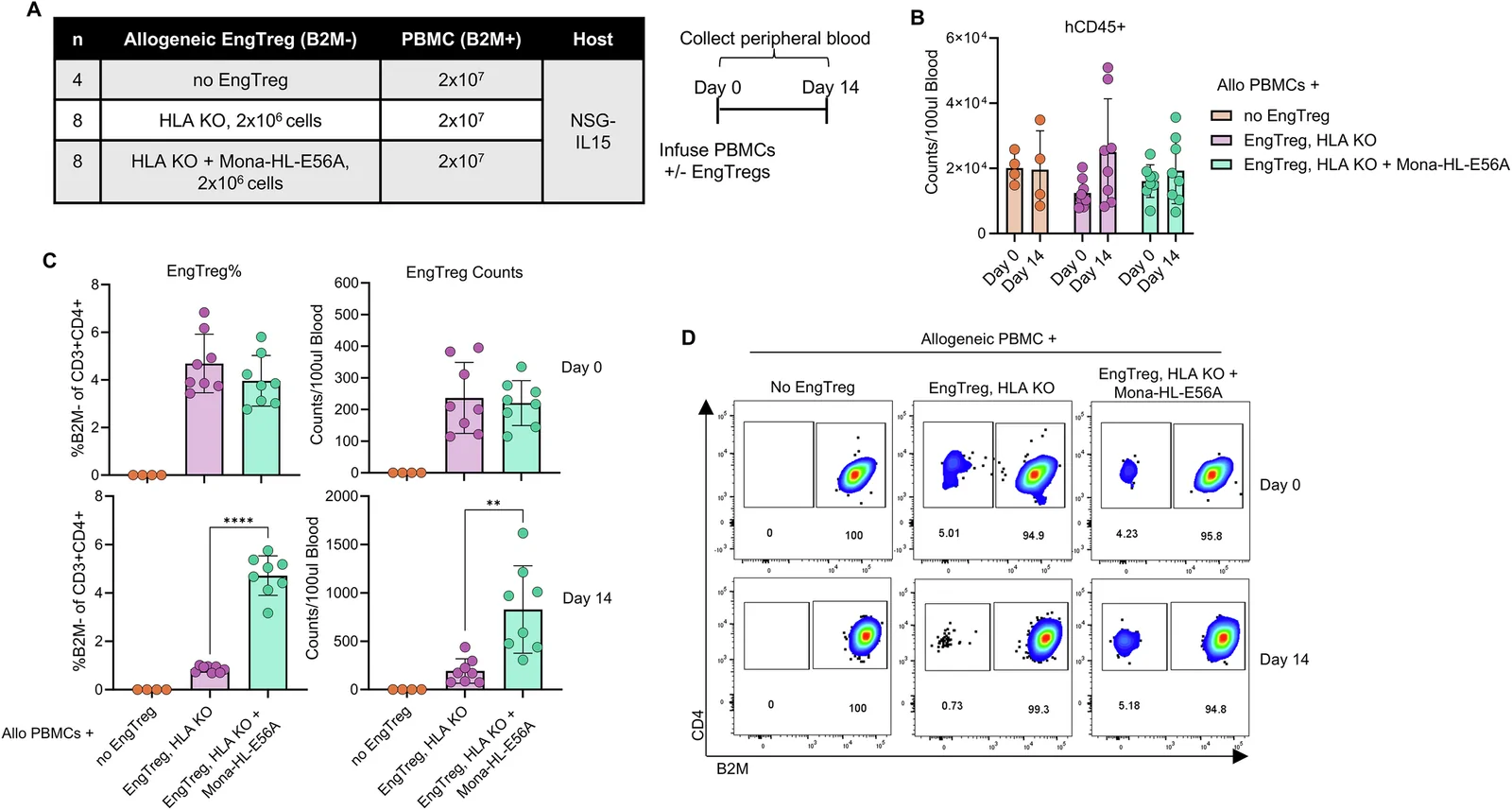
Improved NKG2A engager enhanced persistence of HLA KO allogeneic EngTregs in PBMC-humanized NSG-IL15 mice. (**A**) Tabulated experiment groups with indicated sample size (*n*) and infusion products. PBMC donor is allogeneic to EngTregs and are distinguished based on surface B2M expression. (**B**) Total human CD45 (hCD45) counts in the peripheral blood of animals were quantified on day 0 after infusion and on day 14. (**C**) Frequency among CD4 T cells (left) and counts (right) of B2M- EngTregs were quantified on day 0 and 14. **p < 0.01, ****p < 0.0001 by unpaired *t*-test. Error bars denote mean ± SD. (**D**) Representative flow plots for data shown in panel (**C**). Source data are available online for this figure.

To determine the potential for long-term persistence, a CD34+ hematopoietic stem cell (HSC) humanized NSG-IL15 model was used to capture a robust human lymphoid compartment without the study duration constrained by the onset of xenogeneic graft vs host disease (GvHD) (Kagoya et al, 2020). Here, allogeneic HLA replete, HLA KO, or HLA KO with engager EngTregs (Appendix Fig. S1A) were infused to animals 12-weeks post HSC humanization (Fig. 5A), when substantial human immune cells have reconstituted the animals (Abeynaike et al, 2023; Aryee et al, 2022). All infusion products were marked with transgenic LNGFR for tracking—HLA replete and KO cells were transduced with an LNGFR-only vector, whereas HLA KO with engager cells were transduced with a vector co-expressing LNGFR and Mona-HL-E56A engager. The NKG2A engager-encoding vector was transduced at a higher efficiency than the LNGFR-only vector (Appendix Fig. S1A). To avoid excessive differences in the LNGFR+ cell dose in each group, we compensated for the differences in LNGFR+ frequency by dosing less total EngTregs in the NKG2A engager group (Fig. 5A). Persistence of EngTregs was tracked over the course of 12 weeks post-infusion. An untreated group served as a negative control for LNGFR detection (Fig. 5A). While all EngTreg products were detectable after infusion on day 0 in the peripheral blood, only HLA KO + Mona-HL-E56A EngTregs were detectable after day 20 and continued to persist for the duration of the 12-week experiment (Fig. 5B; Appendix Fig. S1B,C).

**Figure 5:**
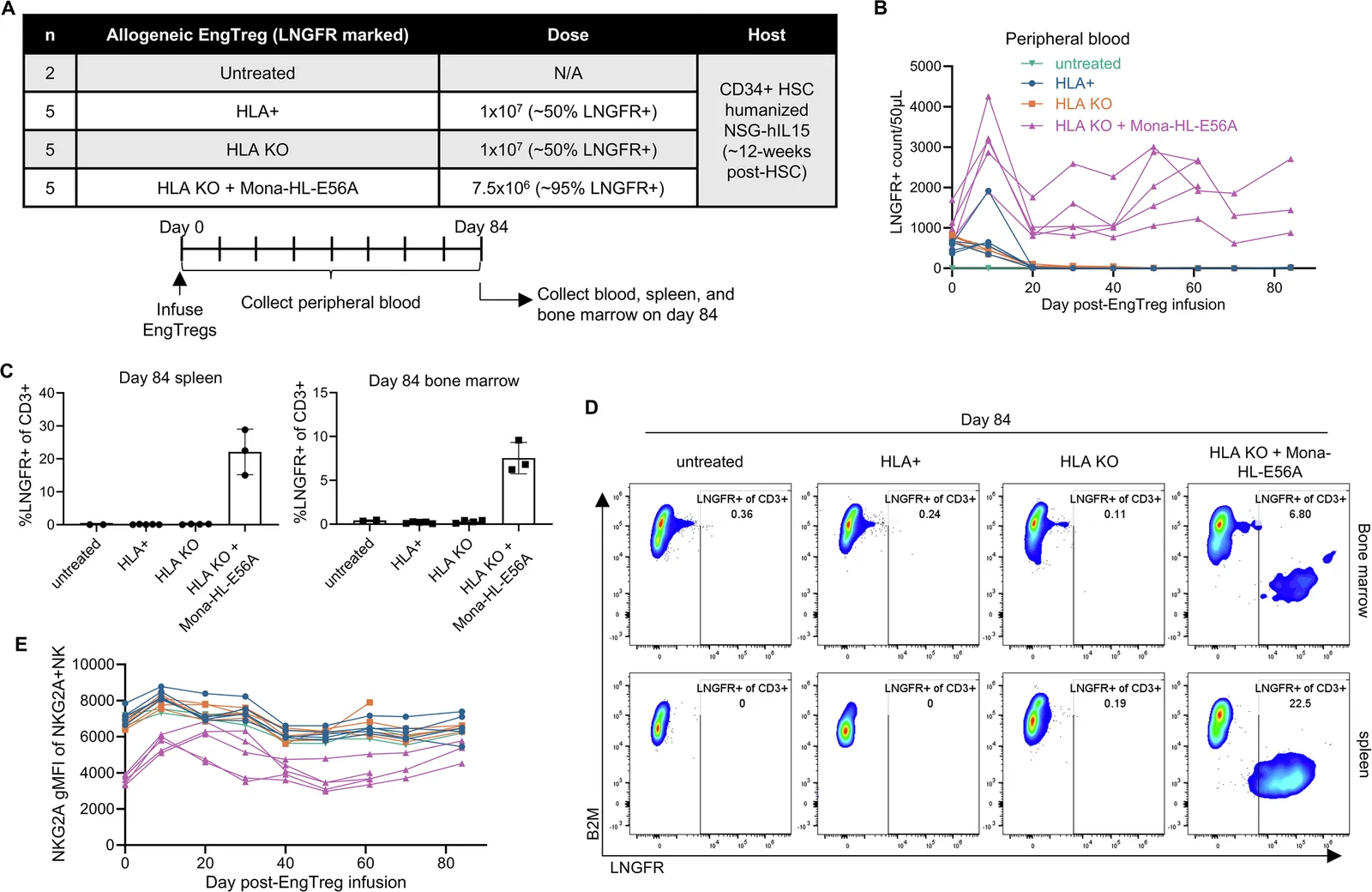
HLA KO and expression of improved NKG2A engager enabled months-long persistence of allogeneic EngTregs in CD34+ HSC-humanized NSG-IL15 mice. (**A**) Tabulated experiment groups with indicated sample size (*n*) and infusion products. CD34+ HSC donor is allogeneic to LNGFR-marked EngTregs, which were infused 12 weeks post HSC humanization. (**B**) Frequency of LNGFR+ cells in the peripheral blood were quantified from day 0 post-infusion to day 84 (12 weeks). (**C**) Animals were sacrificed on day 84, and the frequency of LNGFR+ cells in the spleen and bone marrow were quantified. Error bars denote mean ± SD. *n* = 2, 5, 4, 3 for the Untreated, HLA+, HLA KO, and HLA KO+Mona-HL-E56A groups, respectively, at the final timepoint analysis. (**D**) Representative flow plots for the data shown in panel (**C**). (**E**) Geometric mean fluorescence intensity (gMFI) of NKG2A expression on peripheral blood NKG2A + NK cells were quantified over time. Each line represents one animal. Source data are available online for this figure.

No remarkable differences were observed in major human immune subsets across the groups (Fig. EV4A). Several animals were sacrificed due to humane endpoint in both the HLA KO and HLA KO + engager groups around day 60 (Fig. EV4B), consistent with the occasional morbidity observed in HSC-humanized NSG-IL15 mice on this timescale (Aryee et al, 2022). On day 84 (end of week 12), surviving animals were sacrificed, and the presence of LNGFR+ T cells was measured in the spleen and bone marrow. Similar to peripheral blood, LNGFR+ cells were only observed in the HLA KO + Mona-HL-E56A group (Fig. 5C–D). These data demonstrate that HLA replete and HLA KO allogeneic cells fail to persist—as expected with T cell and NK cell-mediated rejection, respectively—whereas expression of the Mona-HL-E56A NKG2A engager in combination with HLA KO enables months-long persistence of allogeneic EngTregs in the HSC-NSG-IL15 model. Interestingly, a modest but consistent downregulation of surface NKG2A among NKG2A+ NK cells was observed among engager-expressing EngTreg-treated animals relative to other groups (Fig. 5E), which is aligned with the NKG2A engager interacting with its cognate target in vivo. Together, these two humanized models demonstrate that the Mona-HL-E56A engager enables long-term persistence of HLA KO EngTregs in allogeneic settings in vivo.

**Figure EV4:**
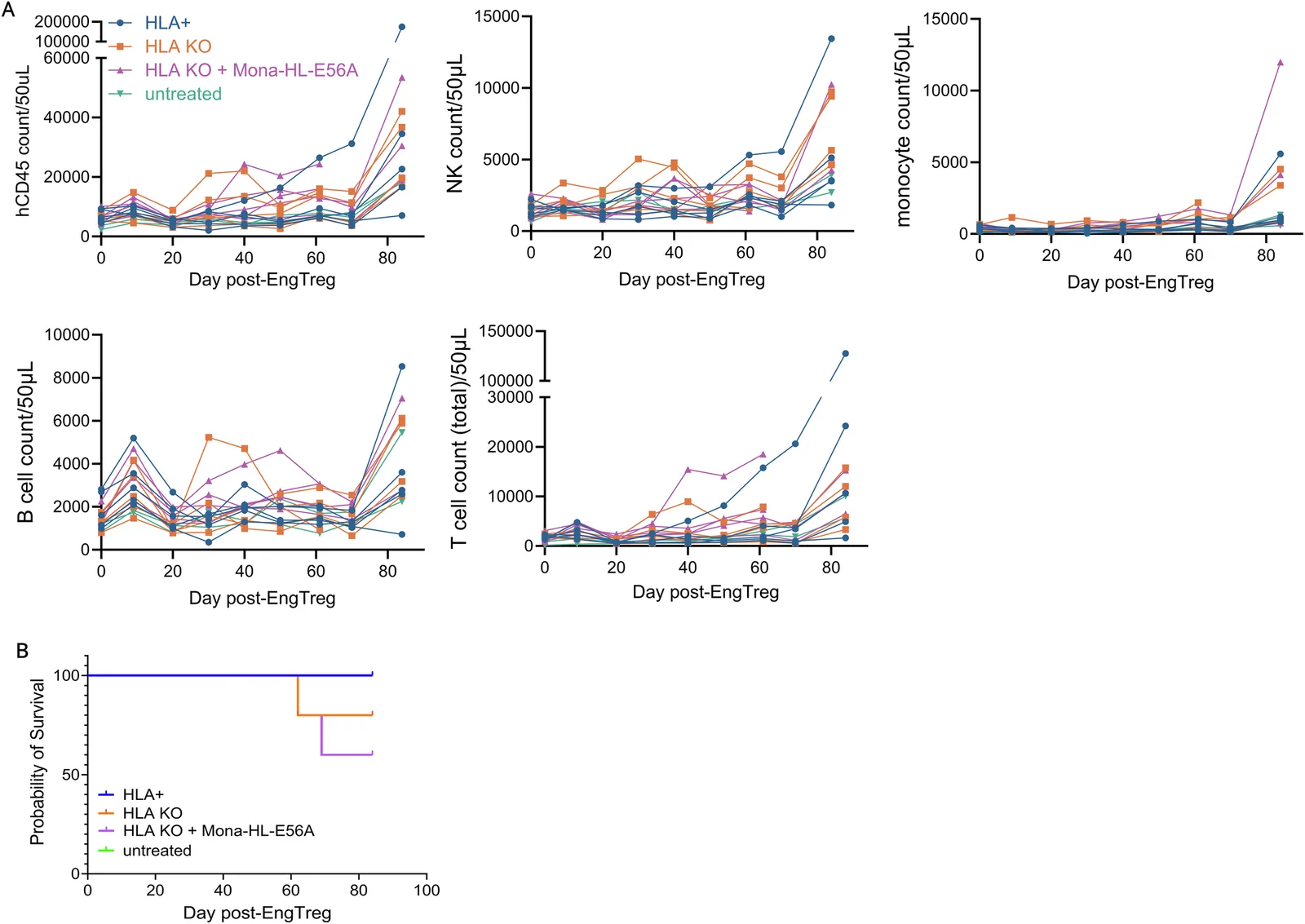
Peripheral immune subsets and survival over time for HSC-humanized mice. (**A**) Plots for quantifying the indicated immune population in the peripheral blood of animals in Fig. 5. Each line denotes one unique animal. (**B**) 1 and 2 animals in the HLA KO and HLA KO + Mona-HL-E56A group, respectively, were sacrificed at the indicated times based on Body Condition Score. Sample sizes for each group were the same as described in the legend of Fig. 5.

### Improved NKG2A engager readily incorporates into a clinically applicable EngTreg product

A clinical-stage autologous EngTreg (GNTI-122) has been developed to treat Type 1 Diabetes (T1D)(Uenishi et al, 2024). GNTI-122 is manufactured using a dual-AAV knock-in approach to generate stable FOXP3+ regulatory T cells, composed of constitutively high and stable FOXP3 expression (Honaker et al, 2020), a heterodimeric chemically inducible signaling complex (CISC) that delivers rapamycin-dependent IL-2 signals(Cook et al, 2023), and the pancreatic islet autoantigen-specific IGRP305-T cell receptor (TCR) (Yang et al, 2022). As a next-generation off-the-shelf allogeneic EngTreg product for T1D, we report GNTI-148, where HLA KO and our improved NKG2A engager are incorporated into our established dual site-specific knock-in platform. In short, CD4+ T cells from healthy donors are stimulated and electroporated with four CRISPR/Cas9 ribonucleoprotein complexes targeting the *FOXP3*, *TRAC*, *B2M*, and *CIITA* loci. Following electroporation, two AAVs are transduced to deliver site-specific knock-in templates: (1) a vector encoding the MND promoter, a CISC subunit, and Mona-HL-E56A engager is introduced upstream of the endogenous *FOXP3* gene; (2) another AAV encoding MND, other half of CISC, and the Vβ13.6+ islet antigen-specific IGRP305-TCR is knocked-in at *TRAC*, whereby the heterologous IGRP305-TCR hijacks the endogenous TCRα constant domain (Fig. 6A). Subsequent culture in the presence of rapamycin elicits IL-2 signaling by dimerizing CISC subunits, resulting in enrichment of dual-vector engineered cells (Cook et al, 2023; Uenishi et al, 2024).

**Figure 6:**
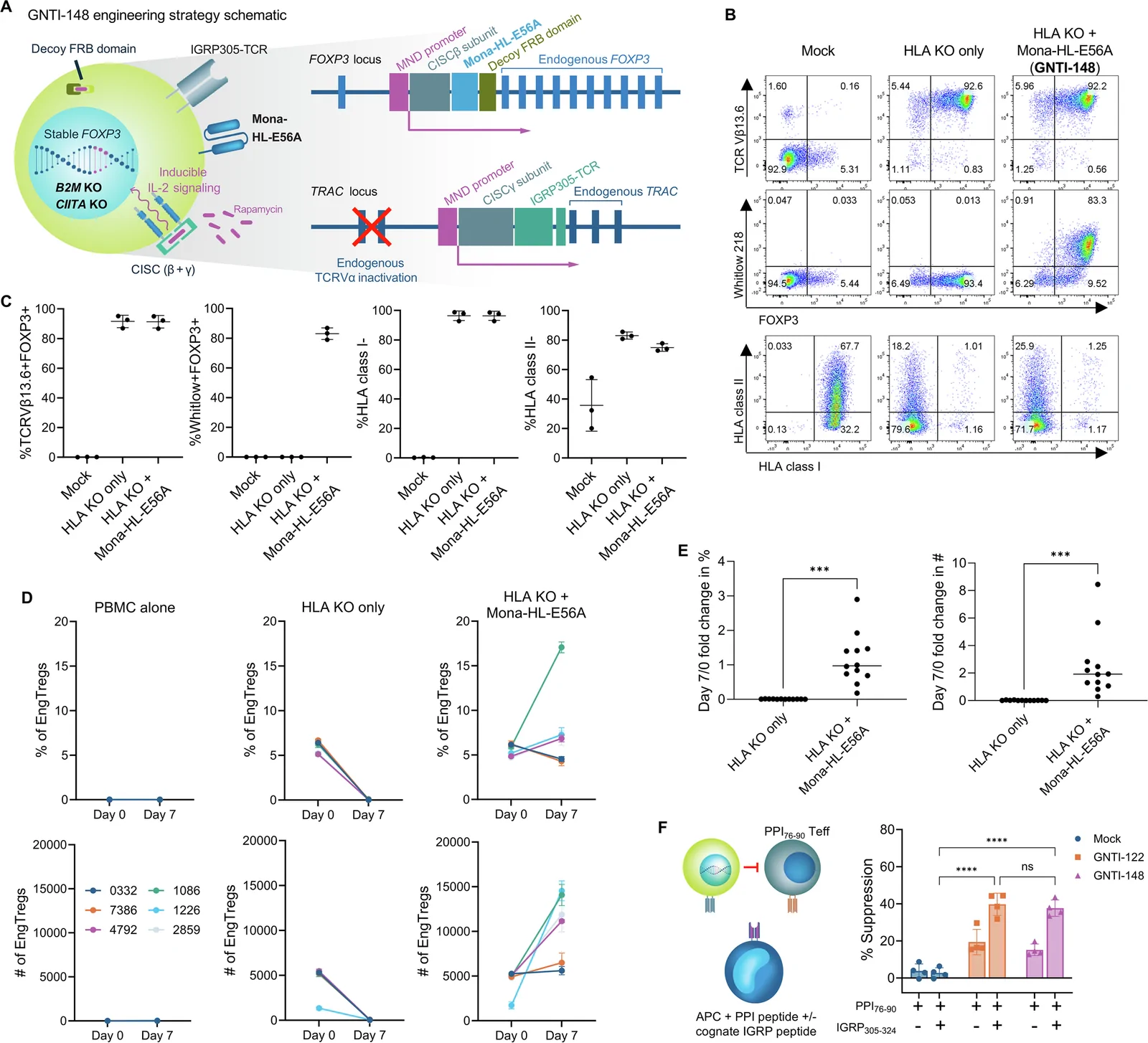
GNTI-148, an HLA KO with improved NKG2A engager allogeneic EngTreg asset for treating type 1 diabetes. (**A**) Schematic depicting the composition of GNTI-148, which is knocked-out for *B2M* and *CIITA*, with the Mona-HL-E56A engager expressed upstream of FOXP3 under a strong promoter and an islet-autoantigen- specific T cell receptor (IGRP305-TCR) expressed from the *TRAC* locus. A heterodimeric chemically inducible signaling complex (CISC) is split across the *FOXP3* and *TRAC* loci to enable enrichment of dual-edited cells via culture with rapamycin. An additional decoy FRB domain sequesters excess rapamycin to prevent inhibition of mTOR signaling in EngTregs. (**B**) Representative phenotype gated on CD4+ cells of mock-engineered CD4 T cells, GNTI-148 without the engager but otherwise identical (HLA KO only), and GNTI-148 (HLA KO + Mona-HL-E56A) research-grade products are shown. (**C**) Frequencies of IGRP305-TCR+ (Vβ13.6+ ) and FOXP3+ dual-knock-in, Mona-HL-E56A engager+ (Whitlow 218+) FOXP3+, and HLA class I and II ablated cells were quantified in each cell product generated from three donors (*n* = 3). (**D**) Frequency (top) and cell number (bottom) of GNTI-148 and control HLA KO only EngTreg products in co-culture with 6 allogeneic PBMCs were quantified on day 0 and 7 as described in Fig. 3C. EngTregs were detected by P2A+ Vβ13.6+ gated on the live CD3+ CD4+ subset. Data were representative of two donor sources of EngTregs as target cells. *n* = 6 PBMC responders, with each condition tested in technical triplicate. (**E**) Fold change in frequency (left) or cell number (right) from day 7 over day 0 for the indicated EngTreg target cells. *n* = 12, six allogeneic PBMC responders tested against two batches of EngTregs from unique donors. Bars denote median. ***p < 0.001 by paired *t*-test or Wilcoxon test for change in % and #, respectively. (**F**) Schematic depicting the assay system is shown on the left. Suppression of a bystander autoantigen-specific effector T cell (Teff) was measured for each indicated cell product. DRB1*04:01+ antigen-presenting cells (APCs) were autologous to the Teff. *n* = 4, with two donor sources of suppressors co-cultured against two donor sources of Teff. ns *p* > 0.05, ****p < 0.0001 by two-way ANOVA with Bonferroni’s multiple comparisons test. Error bars denote mean ± SD in all panels. Source data are available online for this figure.

After production, a similar purity of FOXP3+ Vβ13.6+ cells was observed with the GNTI-148 vectors and the first-generation system without an engager (HLA KO only) (Fig. 6B,C). Mock unedited CD4+ T cells expanded alongside the EngTregs served as a control for gene-edited and Treg-associated phenotypic markers. Encoding the engager did not impact expansion (Fig. EV5A) or Treg-associated phenotype (Fig. EV5B) of GNTI-148. Engager expression was detectable only in GNTI-148 and not in control cells, and surface HLA class I and class II were successfully ablated (Fig. 6B,C). Importantly, GNTI-148 cells persisted in the 7-day allogeneic PBMC co-culture rejection system, whereas HLA KO only EngTregs did not (Figs. 6D,E and EV5C). In an antigen-specific bystander suppression assay, GNTI-148 effectively suppressed autoantigen-specific effector T cells targeting a preproinsulin-derived epitope (PPI_76–90_) (Yang et al, 2022). The potency of suppression was comparable with that of donor-matched GNTI-122 (Figs. 6F and EV5D), which do not possess HLA KO or Mona-HL-E56A engager expression for immune evasive engineering. These results demonstrate the functionality of the Mona-HL-E56A engager in the dual-AAV format for generating durable allogeneic EngTreg therapies, without observable impact on immunomodulatory functions.

**Figure EV5:**
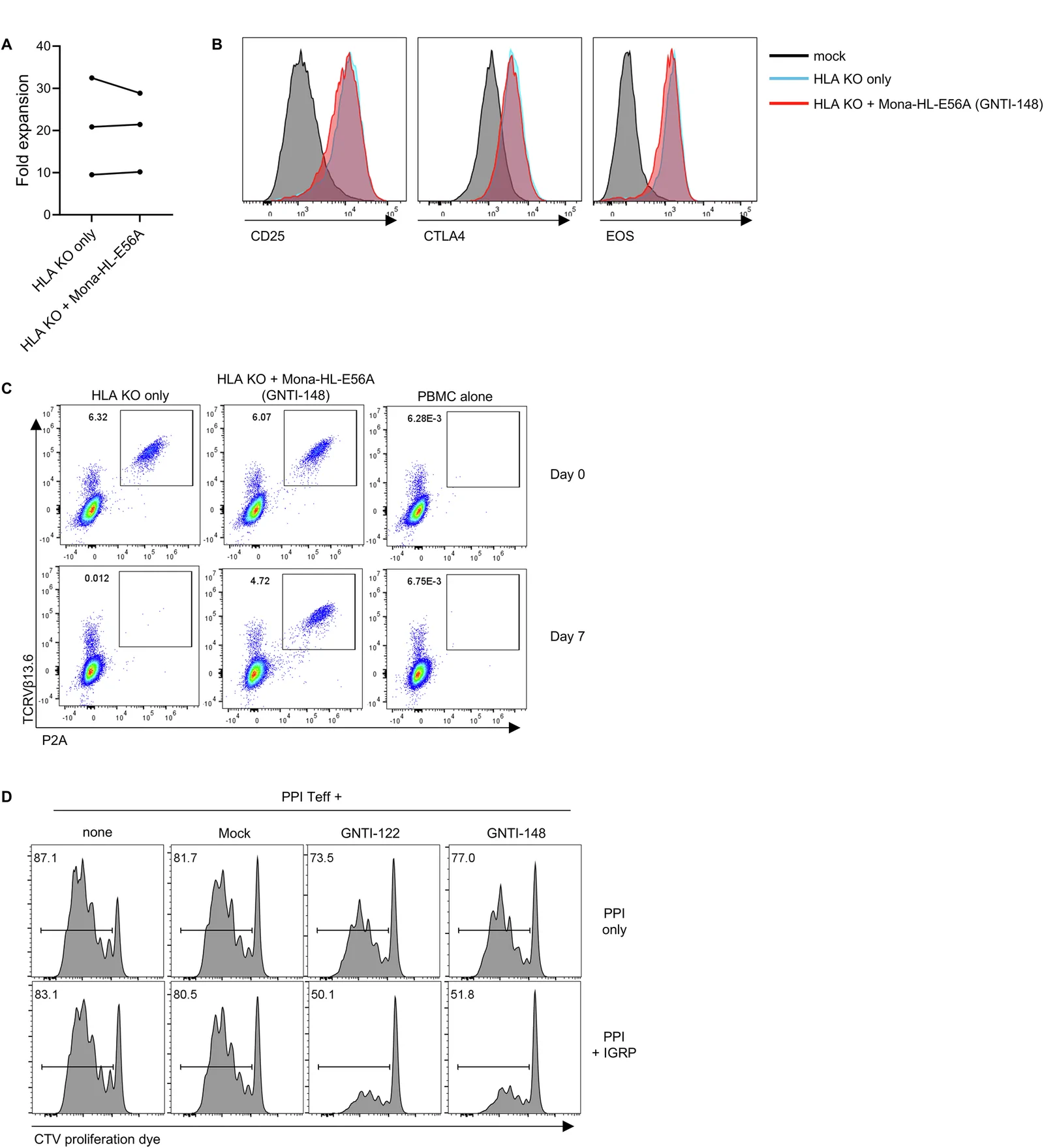
Phenotype and function of GNTI-148 cells. (**A**) Fold expansion of research-grade control (HLA KO only) or GNTI-148 (HLA KO + Mona-HL-E56A) EngTregs from day 7 (start of rapamycin) to day 21 (cryopreservation). Each line denotes a unique donor (*n* = 3). (**B**) Representative flow plots gated on live CD4+ cells showing expression of regulatory T cell-associated markers. (**C**) Representative flow plots gated on live CD3+ CD4+ cells for quantifying EngTreg presence in co-cultures shown in Fig. 6D, E. (**D**) Representative flow plots gated on CTV+ CFSE- effector T cells for quantifying % suppression shown in Fig. 6F.

## Discussion

Robust NK inhibition to prevent HLA KO allogeneic cell rejection is an active area of investigation in the immune evasion field. Here, we reported an improved NKG2A engager as a synthetic ligand for robust inhibition of human NK cell rejection of HLA KO target cells. The NKG2A engager overcomes the limitation of the natural ligand, HLA-E, with inhibitory but also stimulatory effects that would instigate rejection by NKG2C+ NK cells. The efficacy of the engager was dependent on the choice of NKG2A-binding clone, and likely the affinity of the interaction—the precise molecular mechanisms by which these parameters influence function warrant further investigation. In addition to engaging NKG2A, synthetic engager approaches have recently been applied to other inhibitory NK receptors, such as SIRPα, TIM3 (Gravina et al, 2023), CD300A (Zhang et al, 2025), and inhibitory KIRs (Hiura et al, 2025). Notably, TIM3 and CD300A exemplify how a synthetic engager is advantageous over the native ligand(s): CD300A binds phospholipids (Simhadri et al, 2012), which are not readily controllable in cells, and TIM3 binds to proteins with pleiotropic effects, including galectin-9, HMGB1, and CEACAM1 (Das et al, 2017). Therefore, synthetic engagers broaden the repertoire of engineerable ligands, and could be additive with native ligands, to control host cell immune responses.

Beyond the HLA KO with NK inhibitor approach, other immune evasion strategies have been developed. Mo et al described an alloimmune defense receptor (ADR), which is a 41BB-targeted chimeric antigen receptor (CAR) (Mo et al, 2021). 41BB is upregulated on activated lymphocytes, thereby allowing an allogeneic cytotoxic lymphocyte engineered with ADR to eliminate host alloreactive immune cells. Due to the cytotoxicity-dependent mechanism of ADR, it is not a universal solution and cannot be applied to allogeneic cell therapies lacking cytotoxic functions. In contrast to transgenic expression of immune evasion molecules, multiple groups have shown that knockout of activating molecules on HLA KO allogeneic cells can reduce NK responses. Ablating CD155, a ligand for the activating receptor DNAM-1, mitigated rejection of HLA KO induced pluripotent stem cells (iPSCs) by NK cells (Chimienti et al, 2022; Wang et al, 2021). Knockout of adhesion molecules, CD54 and CD58, required to form an effective immune synapse, also reduced NK responses against HLA KO iPSC targets (Hammer et al, 2024). Lastly, HLA knockdown, rather than knockout, has been explored to retain sufficient HLA class I for NK inhibition while reducing T cell-mediated alloresponses (Golinelli et al, 2025; Grauwet et al, 2024; Perica et al, 2025). Direct side-by-side comparison using rigorous preclinical models could delineate the relative efficacy of the plethora of immune evasion approaches.

In this study, we employed a series of in vitro and in vivo rejection models to investigate the effect of immune evasion technologies. While a short-term (hours to a few days) co-culture between immune responders and targets is commonly used (Grauwet et al, 2024; Gravina et al, 2023; Hiura et al, 2025; Zhang et al, 2025), the week-long co-culture using total PBMCs at a high effector:target ratio presents a rigorous threshold for target cell persistence. T and NK cells are present for the duration of the co-culture in the PBMC system and can measure both HLA-targeted and missing-self rejection mechanisms. Without needing to purify specific subsets, the assay captures the core biology while enabling higher throughput for assessing multiple test articles and responder donors in parallel. In a preclinical in vivo setting, assessment of human allorejection responses relies on humanized mouse models. Whether using PBMC or HSC as source of humanization, using human IL-15 transgenic immunodeficient mice as the host supports robust assessment of human NK responses. Without the human *IL15* transgene, adoptive transfer of PBMC-derived NK cells engrafted poorly in immunodeficient mice (Coholan et al, 2025; Katano et al, 2017), and human NK cells develop poorly in terms of number and phenotype after HSC engraftment (Abeynaike et al, 2023; Aryee et al, 2022). Through a combination of robust in vitro and appropriate in vivo models, HLA-targeted T cell alloreactivity and NK-mediated missing-self rejection can be effectively studied preclinically in the human system.

Although we identified NKG2A as a potent axis for inhibiting missing-self rejection by human NK cells, inhibition through NKG2A was not sufficient for protecting HLA-deficient cells against a minor subset of PBMC responders (Fig. 3C). Given the contribution of inhibitory KIRs to human NK cell licensing, using engagers to target inhibitory KIRs is a valuable approach. A pan-inhibitory KIR binder without cross-reactivity to activating KIRs has been difficult to generate historically (Vey et al, 2018), but antibody engineering advancements could provide the necessary solutions, as demonstrated by the recent work of Hiura and colleagues in identifying binders selective for the inhibitory but not activating KIRs. A combination of three engagers was able to cover KIR2DL1, KIR2DL2/3, and KIR3DL1 engagement (Hiura et al, 2025), collectively representing the repertoire of key inhibitory KIRs that license human NK cells (Fauriat et al, 2008). Importantly, the group observed that a NKG2A-selective engager based on a different clone than Mona-HL-E56A provided minimal protection to B2M KO iPSCs challenged with certain NKG2C-high NK samples in vitro, whereas combining that engager with an inhibitory KIR engager improved protection in the same context (Hiura et al, 2025). Combining the improved NKG2A engager described here with engagers specific for inhibitory KIRs is a potential path for improving the breadth of immune evasion across diverse NK responders.

Given the known contribution of KIR-licensed NK cells to missing-self rejection, the lack of significant associations between any of the inhibitory KIR+ NK cell frequencies and the magnitude of B2M KO target elimination was an interesting finding in this study (Fig. EV1F). We speculate on three hypotheses underlying this observation: (1) In the case of KIR2DL1+ NK cell detection, the HP-MA4 clone used here binds to both KIR2DL1 and activating KIR2DS1/S3/S5. Characterization using a more specific reagent or a combination of detection antibodies will accurately delineate the frequency of KIR2DL1+ NK cells (Le Luduec et al, 2019; Meazza et al, 2022), which may result in a stronger correlation with missing-self killing; (2) Unlike NKG2A+ NK cells, which are licensed in all individuals due to ubiquitous HLA-E expression, inhibitory KIR+ NK cells are only licensed if appropriate HLA class I is also encoded in that individual, leading to more consistent missing-self responses by NKG2A+ NK cells; and (3) NKG2A-licensed human NK cells may simply be more cytotoxic than KIR-licensed cells against HLA-deficient targets, thereby contributing more to the missing-self response. Multiple independent groups have reported higher cytotoxicity and/or activation of human NKG2A+ NK cells versus other NK subsets when stimulated with B2M KO primary T cells (Hammer et al, 2024; Perica et al, 2025) or K562 (Highton et al, 2020; Rathmann et al, 2017; Sim et al, 2016). In particular, Altfeld and colleagues also found that NKG2A+ human NK cells experience higher peak calcium signaling than NKG2A-negative cells when stimulated through activating receptors (Highton et al, 2020). These mechanisms in isolation or in concert could explain the observed weaker relationship between inhibitory KIRs, compared to NKG2A, and missing-self rejection.

In conclusion, we report NKG2A as a potent axis for human NK inhibition when selectively engaged using a membrane-anchored binder. HLA KO EngTregs expressing an improved NKG2A engager demonstrated robust persistence in vitro and in vivo under allogeneic settings that rejected unprotected cells. Our development potentiates effective off-the-shelf allogeneic EngTregs to treat autoimmune diseases through products such as GNTI-148 described here. Beyond EngTregs, other allogeneic cell types may also benefit from the Mona-HL-E56A NKG2A engager to achieve durable clinical effects to overcome the limitations of personalized autologous therapies.

## Methods

**Table:** Reagents and tools table

Reagent/resource	Reference or source	Identifier or catalog number
**Experimental models**		
NSG-IL15	The Jackson Laboratory	NOD.Cg-Prkdc^scid^ Il2rg^tm1Wjl^ Tg(IL15)1Sz/SzJ (030890)
**Recombinant DNA**		
pALD-Lenti	Aldevron	5030-5
pALD-Rev	Aldevron	5032-5
pALD-VSV-G	Aldevron	5036-5
pALD-GagPol	Aldevron	5034-5
pALD-ITR	Aldevron	5062-10
pALD-HELP	Aldevron	5082-10
pALD-AAV6	Aldevron	5059-10
**Antibodies and flow cytometry reagents**		
Live/dead 7-AAD	BioLegend	420404 (1:25)
Live/dead Near-IR	Thermo Fisher	L10119 (1:1000)
Human TruStain FcX (FcR block)	BioLegend	422302 (1:20)
CellTrace CFSE	Thermo Fisher	C34554
CellTrace Violet	Thermo Fisher	C34557
Cell Proliferation Dye eFluor670	Thermo Fisher	65-0840-90
Mouse anti-human CD14, clone M5E2	BioLegend	301830 (1:70)
Mouse anti-human CD19, clone SJ25C1	BD Biosciences	612917 (1:100)
Mouse anti-human CD3, clone UCHT1	BD Biosciences	563546 (1:100), 612940 (1:200)
Mouse anti-human CD3, clone UCHT1	BioLegend	300464 (1:200)
Mouse anti-human CD56, clone HCD56	BioLegend	318334 (1:100)
Mouse anti-human NKG2A, clone S19004C	BioLegend	375108 (1:100)
Mouse anti-human NKG2C, clone S19005E	BioLegend	375003 (1:50)
Mouse anti-human ILT2, clone GHI/75	BioLegend	333719 (1:200)
Mouse anti-human KIR2DL1/S1/S3/S5, clone HP-MA4	BioLegend	339509 (1:200)
Mouse anti-human KIR2DL2/3, clone DX27	BioLegend	312611 (1:200)
Mouse anti-human KIR3DL1, clone DX9	BioLegend	312715 (1:50)
His-tagged recombinant human NKG2A	ACROBiosystems	NKA-H5244 (1:40)
His-tagged recombinant human NKG2A/CD94	ACROBiosystems	NC4-H52H3 (1:30)
anti-His tag, clone AD1.1.10	Abcam	ab72467 (1:100)
Rabbit anti-Whitlow 218, clone E3U7Q	Cell Signaling Technology	69310 L (1:100)
Mouse anti-human HLA-DR	BioLegend	307614 (1:1000)
Mouse anti-human B2M, clone 2M2	BioLegend	316304 (1:200), 316308 (1:2000), 316312 (1:100)
Mouse anti-human HLA-E, clone 3D12	BioLegend	342605 (1:200)
Mouse anti-human CD47, clone CC2C6	BioLegend	323124 (1:100)
Mouse anti-human LNGFR, clone ME20.4	BioLegend	345106 (1:100), 345110 (1:1000)
Mouse anti-human HLA-ABC, clone W6/32	BioLegend	311404 (1:400)
Mouse anti-human HLA class II, clone Tü39	BioLegend	361708 (1:200)
Rat anti-mouse CD45, clone 30-F11	BioLegend	103128 (1:200)
Rat anti-mouse CD45, clone 30-F11	BD Biosciences	566439 (1:400)
Mouse anti-human CD45, clone HI30	BD Biosciences	612891 (1:200), 568524 (1:200)
Mouse anti-human CD4, clone RPA-T4	BD Biosciences	564724 (1:200)
Mouse anti-human CD14, clone 63D3	BioLegend	367126 (1:100)
Mouse anti-human CD19, clone HIB19	BioLegend	302240 (1:200)
Mouse anti-human CD3, clone SK7	BioLegend	344828 (1:200)
Mouse anti-human CD56, clone NCAM16.2	BD Biosciences	564057 (1:100)
Mouse anti-human B2M, clone A17082A	BioLegend	395706 (1:100)
Recombinant anti-human TCRVβ13.6, clone REA554	Miltenyi Biotec	130-126-523 (1:100)
Mouse anti-human TCR TCRVβ13.6, clone JU74.3	Beckman Coulter	B07225 (1:800)
Mouse anti-human FOXP3, clone 259D	BioLegend	320216 (1:100)
Mouse anti-human CD25, clone M-A251	BD Biosciences	741144 (1:200)
Mouse anti-human CTLA4, clone BNI3	BioLegend	369606 (1:100)
Rat anti-human EOS, clone W16032A	BioLegend	399306 (1:100)
Mouse anti-P2A, clone 3H4	Novus Biologicals	NBP2-59627AF488 (1:800)
Precision Count Beads	BioLegend	424902
CountBright Plus Absolute Counting Beads	Thermo Fisher	C36995
**Oligonucleotides and other sequence-based reagents**		
Recombinant NK inhibitor molecules	Described in “*Constructs*” section of Methods, or from this study	Appendix Table S1
**Chemicals, enzymes and other reagents**		
Anti-biotin Microbeads	Miltenyi Biotec	130-097-046
Human CD4 T Cell Isolation Kit	Miltenyi Biotec	130-096-533
SpyFi Cas9	Aldevron	9214-5MG
Rapamycin	Alfa Aesar	J67452.XF
Dynabeads Human T-Expander CD3/CD28	Thermo Fisher	11141D
Human IL-2	Thermo Fisher	200-02-50UG
RPMI-1640	Thermo Fisher	21870076
GlutaMAX	Thermo Fisher	35050061
2-mercaptoethanol	Thermo Fisher	21985023
HEPES	Thermo Fisher	15630080
Penicillin-streptomycin	Corning	30-002-Cl
Fetal bovine serum	Omega Scientific	FB-11
LV-MAX Lentiviral Production System	Thermo Fisher	A35684
LentiBOOST	Revvity	SB-P-LV-101-12
Opti-MEM	Thermo Fisher	31985062
AAV-MAX Helper-Free AAV Production System	Thermo Fisher	A51217
FectoVIR-AAV	Sartorius	101000004
Benzonase	MilliporeSigma	71206-3
HiTrap Capto AVB	Cytiva	17372211
Cesium Chloride	MilliporeSigma	C3139-1KG
PBS	VWR	75800-984
Foxp3/Transcription Factor Staining Buffer Set	Thermo Fisher	00-5523-00
AP21967	Takara Bio	635055
**Software**		
Graphpad Prism ver10	https://www.graphpad.com/	
FlowJo ver10.8	https://www.flowjo.com/	
**Other**		
4D-Nucleofector	Lonza	
Xenon Electroporation System	Thermo Fisher	
NovoCyte Penteon	Agilent	
FACSymphony A3	BD	
FACSymphony A5	BD	
Cytek Aurora	Cytek Biosciences	

### Constructs

Recombinant DNA sequences were codon optimized for expression in human T cells, synthesized, and cloned at GenScript (Piscataway, NJ) into third-generation lentiviral or AAV packaging plasmids from Aldevron (Fargo, ND). All transgenes were expressed under the MND promoter (Honaker et al, 2020) with a GSG linker, P2A self-cleaving peptide, and a truncated LNGFR marker downstream of the NK inhibitor. Amino acid sequences for the HLA-E*01:03 allele and CD47 from US Patent Pub. No. 2021/0292715A1 were used. The original coding sequence of the CD300A TASR V2 (Zhang et al, 2025) was used in the lentiviral vector. Amino acid sequences for all NK inhibitors tested are listed in Appendix Table S1. AAV vectors used in Fig. 6 were designed as described previously (Uenishi et al, 2024), with a codon-optimized P2A separating each transgene.

### Cells

Healthy donor Leukopak-derived PBMCs were sourced from StemCell Technologies (Vancouver, Canada) or the American Red Cross (Philadelphia, PA) with IRB approval. CD4+ T or NK cells were isolated from PBMC using negative selection kits from Miltenyi Biotec (Bergisch Gladbach, Germany) to generate EngTregs using site-specific knock-in with AAV templates or to be used as effector cells, respectively. As described previously, polyclonal EngTregs were used in Figs. 1–5 were generated by knock-in only at the *FOXP3* locus (Cook et al, 2023), while the antigen-specific EngTregs in Fig. 6 were generated by knock-in at both *FOXP3* and *TRAC* loci (Uenishi et al, 2024). In short, purified CD4+ T cells were stimulated on day 0 for three days using CD3/CD28 Dynabeads and 50 ng/mL IL-2 (Thermo Fisher, Waltham, MA) and electroporated with SpyFi Cas9 (Aldevron) complexed with single guide RNAs (sgRNAs; BioSpring, Frankfurt, Germany) targeting *FOXP3* and/or *TRAC* using the 4D-Nucleofector (Lonza, Basel, Switzerland) or Xenon Electroporation System (Thermo Fisher) on day 4. Immediately following electroporation, cells were transduced with AAV vectors. Edited cells on day 7 are then enriched by culturing in 10 nM rapamycin (Thermo Fisher) supplemented every 2–3 days, which stimulates CISC for orthogonal IL-2 signaling, up to day 18-21 with an anti-CD3/CD28 restimulation on day 11. HLA KO was performed using sgRNAs from IDT (Coralville, Iowa) targeting *B2M* and *CIITA* (Kagoya et al, 2020) concurrently with *FOXP3* editing. With the exception of EngTregs in Fig. 6, cells were depleted for surface B2M (clone 2M2) and HLA-DR (clone L243) on day 11 prior to restimulation using biotinylated antibodies from BioLegend (San Diego, CA) and anti-biotin microbeads (Miltenyi Biotec). All cells were cultured in RPMI-1640 media supplemented with GlutaMAX, 2-mercaptoethanol, HEPES (Thermo Fisher), penicillin-streptomycin (Corning, Corning, NY), and 20% fetal bovine serum (Omega Scientific, Tarzana, CA), and at 37 °C with 5% CO_2_.

### Lentiviral vector production and transduction

Third-generation, self-inactivating (SIN) lentiviral vectors were produced via LV-MAX Lentiviral Production System (Thermo Fisher) according to the manufacturer’s protocol. Briefly, LV-MAX suspension 293 cells were cultured in LV-MAX Production Medium and transfected in shaker flasks using the LV-MAX Transfection Reagent with a four-plasmid system encoding the transfer vector, packaging genes, and VSV-G envelope. Cultures were maintained at 37 °C with 5% CO₂ and 125 rpm orbital shaking. Viral supernatants were harvested 72 h post-transfection, clarified by 0.2-μm filtration, and concentrated through ultracentrifugation at 90,000×*g* for 90 min at 4 °C in a swinging-bucket SW32 Ti rotor (Beckman Coulter, Brea, CA). Pelleted virus was resuspended in Opti-MEM (Thermo Fisher) and stored at −80 °C in single-use aliquots until use.

Lentiviruses were functionally titered by transducing Jurkat cells (ATCC, Manassas, VA) at limiting dilutions in a 96-well plate supplemented with a 100-fold dilution of LentiBOOST Solution B (Revvity, Waltham, MA). The lowest dilution that yielded <30% transduction based on LNGFR positivity was used to back calculate the functional titer. EngTregs were transduced with lentivirus at a multiplicity of infection (MOI) of 20, supplemented with LentiBOOST Solution B, 1 day after restimulation.

### AAV vector production and transduction

Recombinant AAV6 vectors were produced using the AAV-MAX Helper-Free AAV Production System (Thermo Fisher), with customized downstream processing. Suspension-adapted AAV-MAX HEK293 cells were cultured in Viral Production Medium and transfected using FectoVIR-AAV (Sartorius, Göttingen, Germany) with a three-plasmid system encoding the ITR-containing transfer vector, AAV2/6 Rep/Cap, and adenoviral helper plasmid. Cultures were maintained in shaker flasks at 37 °C, 8% CO₂, and 125 rpm. At 72 h post-transfection, cells and media were harvested and subjected to a freeze-thaw cycle prior to chemical lysis with AAV-MAX lysis buffer with Benzonase (MilliporeSigma, Burlington, MA) to degrade residual nucleic acids. Lysates were clarified by centrifugation and sterile-filtered through a 0.2-μm PES membrane before loading onto an ÄKTA chromatography system equipped with a HiTrap Capto AVB affinity column (Cytiva, Marlborough, MA). Eluted AAV particles were further enriched by cesium chloride (CsCl) gradient ultracentrifugation at 55,000 rpm overnight at 20 °C in a 70.1 Ti fixed-angle rotor (Beckman Coulter). CsCl was removed by stepwise dialysis into PBS containing 0.001% Pluronic F-68. Final products were aliquoted and stored at −80 °C.

AAVs were titered using Stunner (Unchained Labs, Pleasanton, CA) and transduced to primary CD4+ T cells at an MOI of 5 × 10^4^ and 2.5 × 10^4^ vg/cell for the *FOXP3* and *TRAC* targeted vector, respectively. Immediately after electroporation, CD4+ T cells are transferred to serum-free RPMI-1640 media and incubated with the AAVs at 37 °C for 2 h at 2–5 × 10^6^ cells/mL density in the presence of 50 ng/mL IL-2. After incubation, cells were cultured in complete media at 5 × 10^5^/mL for expansion in G-Rex vessels (Wilson Wolf Manufacturing, Saint Paul, MN).

### Flow cytometry

Cells were pelleted, washed with PBS, and stained for viability and surface markers at 4 °C for 30 min. When staining with His-tagged recombinant human NKG2A (ACROBiosystems, Newark, DE), cells were incubated at 4 °C for 60 min, then washed twice prior to staining with secondary antibody at 4 °C for 30 min. After surface staining and, where applicable, cells were fixed, permeabilized, and stained for intracellular markers using the eBioscience FOXP3 Transcription Factor Staining Buffer Set (Thermo Fisher) per manufacturer’s protocol. The list of detection reagents can be found in the Reagents and tools table. Data were collected using the NovoCyte Penteon (Agilent, Santa Clara, CA), FACSymphony A3 or A5 (BD, Franklin Lakes, NJ), or Cytek Aurora (Cytek Biosciences, Fremont, CA) flow cytometer. A single flow cytometer was consistently used to collect all data within the same experiment. Cell numbers were quantified in flow cytometry using Precision Count Beads (BioLegend) or Invitrogen CountBright Absolute Counting Beads (Thermo Fisher Scientific).

### In vitro rejection assays

For 1-day rejection assays shown in Figs. 1 and 2, target cells were labeled with 1 µM CFSE or CTV (Thermo Fisher) and co-cultured with PBMC responders at a 40:1 (3 × 10^5^:7.5 × 10^3^ cells) ratio of PBMC:EngTreg in 96-well U-bottom plates. Percent dead among labeled cells were quantified using the 7-AAD viability dye (BioLegend) by flow cytometry. In the 3-way co-culture in Fig. 2, cells were cultured at a 10:1:1 ratio (1 × 10^5^:1 × 10^4^:1 × 10^4^) of purified NK:target 1:target 2 in 96-U-bottom plates for 18 h. For the 7-day rejection assays shown in Figs. 3 and 6, EngTregs were co-cultured with autologous or allogeneic PBMCs at a 30:1 (3 × 10^5^:1 × 10^4^ cells) or 60:1 (3 × 10^5^:5 × 10^3^ cells) ratio of PBMC:EngTreg for initial assay development in Fig. 3B, then at a 40:1 (3 × 10^5^:7.5 × 10^3^ cells) ratio for the main assay in Figs. 3C and 6D in 96-well U-bottom plates. Identical sets of co-cultures were prepared for read-out at respective timepoints. All co-cultures were supplemented with 50 ng/mL of IL-2 upon initial set-up.

### PBMC-humanized NSG model

Male NSG-IL15 mice (Jackson Laboratory, Bar Harbor, ME) at 7-weeks of age were co-infused on day 0 with a mixture of PBMCs (2 × 10^7^) and HLA-KO EngTregs (2 × 10^6^, with or without Mona-HL-E56A expression) via intravenous tail vein administration. Responder cells and EngTregs were formulated separately in PBS and mixed immediately prior to dosing. EngTregs were allogeneic to the PBMC donor. Peripheral blood was collected by submandibular bleed 30 min after infusion on Day 0 and again on Day 14 for flow cytometric analysis. Precision Count Beads (BioLegend) were included during sample acquisition for accurate cell count analysis, and numbers were normalized per 100 μl volume to account for instances of lower collection yields. Human CD45+ counts were analyzed within the overall live cell compartment, and frequency and counts of B2M- EngTregs were quantified within the live hCD45+ hCD3+ hCD4+ compartment. All animal studies were conducted in compliance with IACUC-approved protocols.

### CD34 + HSC-humanized NSG model

Female NSG-IL15 mice were humanized with CD34+ HSC from one donor at 4 weeks of age (Jackson Laboratory, Sacramento, CA). Twelve weeks after HSC humanization, animals were used for allogeneic EngTreg dosing. On day −1, in a single enrollment, mice were randomized and distributed into groups to provide an approximately equal body weight average with approximately equal standard deviation differences in each group. After administering EngTregs on day 0, approximately 75 µL peripheral blood was collected via the retro-orbital route on days 0, 9, 20, 30, 40, 50, 61, and 70. One mouse in the HLA KO group was euthanized early on day 62, and two mice in the HLA KO + Mona-HL-E56A group were euthanized early on days 62 and 65 due to a body condition score of 2. On day 84, the surviving animals were euthanized by CO_2_ asphyxiation, and blood, spleen, and bone marrow were collected. To support the survival of EngTregs in this model, all animals were dosed intraperitoneally with 5 mg/kg of AP21967 (Takara Bio USA, San Jose, CA) three times a week, every other week, for the duration of the study. AP21967 is a rapalog that can activate the CISC molecule to deliver IL-2 signaling in EngTregs (Cook et al, 2023), and unlike rapamycin, has limited interaction with wild-type mTOR (Bayle et al, 2006) that would interfere with allograft immune rejection.

Mice were ear-notched for identification and housed in individually ventilated polysulfone cages with HEPA-filtered air at a density of up to five mice per cage. Cages were changed every 2 weeks. The animal room was lighted entirely with artificial fluorescent lighting, with a controlled 12 h light/dark cycle (6 am to 6 pm light). The normal temperature and relative humidity ranges in the animal rooms were 22–26 °C and 30–70%, respectively. The animal rooms were set to have up to 15 air exchanges per hour. Filtered tap water, acidified to a pH of 2.5 to 3.0, and standard rodent chow were provided ad libitum. Veterinary care and oversight were provided throughout the study. All animal studies were conducted in compliance with IACUC-approved protocols.

### Antigen-specific suppression assay

Mock CD4 T cells, GNTI-148, or GNTI-122 EngTreg suppressors (2 × 10^4^ cells) were co-cultured with conventional CD4 T cells transduced with a PPI-specific TCR(Yang et al, 2022) (2 × 10^4^ cells) and 50 Gy irradiated PBMCs (1 × 10^5^ cells, autologous to the effectors) for 4 days in 96-well U-bottom plates. The IGRP (QLYHFLQIPTHEEHLFYVLS) and PPI (SLQPLALEGSLQKRG) peptides from GenScript were added to cultures at 1 µg/mL where indicated. Suppressors and effectors were labeled with 2.5 µM of eFluor670 and CTV dyes (Thermo Fisher), respectively, for identification and measuring proliferation. Proliferation of effector T cells in the presence and absence of suppressor cells was used to calculate percent suppression as described previously (Yang et al, 2022).

### Statistical and data analysis

Flow cytometry data were analyzed using FlowJo version 10.8 (Ashland, OR). Where applicable, the mean of technical replicates for each condition was used to calculate percent protection as follows: $\left(1-\frac{({ \% {{\rm{dead}}}{inh}}^{+}{{\rm{with}}}\,{{\rm{effector}}}- \% \,{{\rm{dead}}}\,{{inh}}^{+}{{\rm{alone}}})}{({ \% {{\rm{dead}}}{inh}}^{-}{{\rm{with}}}\,{{\rm{effector}}}- \% \,{{\rm{dead}}}\,{{inh}}^{-}{{\rm{alone}}})}\right)\times 100 \%$, where *inh*^+^ are cells expressing an inhibitor, and *inh*^−^ are untransduced control cells. Subtracting the % death of *inh*^+/−^ alone conditions accounts for baseline differences in viability of target cells. All error bars in figures denote one standard deviation. Non-parametric tests were used when at least one sample in the comparison did not meet normality based on the D’Agostino–Pearson test; parametric tests were used in all other instances. All statistical tests were two-sided. *P* values less than 0.05 were considered significant. Exact *p* values can be found in Appendix Table S2. Statistical tests are described in respective figure captions and performed using GraphPad Prism version 10 (Boston, MA). Sample sizes were not predetermined using statistical methods. None of the studies were conducted in a blinded fashion.

## Supplementary information

Peer Review File

Appendix

Source data Fig. 1

Source data Fig. 2

Source data Fig. 3

Source data Fig. 4

Source data Fig. 5

Source data Fig. 6

Expanded View Figures

## Data Availability

This study includes no data deposited in external repositories. The source data of this paper are collected in the following database record: biostudies:S-SCDT-10_1038-S44321-026-00507-4.
