# Supplementary material for: A synthetic NKG2A engager enables long-term persistence of HLA-deficient allogeneic engineered Tregs
Source: EMBO Mol Med. 2026 Aug 27;18(10):3939–60. doi: 10.1038/s44321-026-00507-4 (PMC13658791; doi:10.1038/s44321-026-00507-4)
Supplement: Supplementary file 1 [file 44321_2026_507_MOESM1_ESM.pdf]

EMBO Molecular Medicine

A synthetic NKG2A engager enables long-term persistence of HLA-deficient allogeneic engineered Tregs

Tingxi Guo, Kaya Epstein, Maegan Hoover, Nathan Zammit, Jennifer Yam, Scott Hussell, Sophia Hernandez, Abigail Doherty, Payam Zarin, Martina Sassone-Corsi, Lindsay Webb, Gene Uenishi, Thomas Wickham, and Christopher Moore

Corresponding authors: Tingxi Guo (tim.guo@gentibio.com) , Christopher Moore (Chris.Moore@gentibio.com)

Review Timeline:

Submission Date:	3rd Mar 26
Editorial Decision:	17th Apr 26
Revision Received:	29th Jun 26
Editorial Decision:	6th Jul 26
Revision Received:	23rd Jul 26
Accepted:	3rd Aug 26

Editor: Zeljko Durdevic

Transaction Report:

(Note: With the exception of the correction of typographical or spelling errors that could be a source of ambiguity, letters and reports are not edited. Depending on transfer agreements, referee reports obtained elsewhere may or may not be included in this compilation. Referee reports are anonymous unless the Referee chooses to sign their reports.)

17th Apr 2026

Dear Dr. Guo,

Thank you for the submission of your manuscript to EMBO Molecular Medicine, and please accept my apologies for the unusual delay in getting back to you. We have now received feedback from two of the three reviewers who agreed to evaluate your manuscript. As the referee #3 will unfortunately not be able to return his/her report in a timely manner, we prefer to make a decision now in order to avoid further delay in the process. Should referee #3 provide a report, we will send it to you, with the understanding that we will not ask for an additional revision.

As you will see from their reports pasted below, while the referee #2 supports publication raising important but minor concerns, referee #1 recognizes interest of the study but also raises serious concerns that should be addressed in a major revision. If you would like to discuss further the points raised by the referees, I am available to do so via email or video. Let me know if you are interested in this option.

Further consideration of a revision that addresses reviewers' concerns in full will entail a second round of review. EMBO Molecular Medicine encourages a single round of revision only and therefore, acceptance or rejection of the manuscript will depend on the completeness of your responses included in the next, final version of the manuscript. For this reason, and to save you from any frustrations in the end, I would strongly advise against returning an incomplete revision.

We would welcome the submission of a revised version within three months for further consideration. Please let us know if you require longer to complete the revision.

I look forward to receiving your revised manuscript.

Yours sincerely,

Zeljko Durdevic

Zeljko Durdevic
Senior Editor
EMBO Molecular Medicine

*****

When preparing your revised manuscript, please refer to our guidelines: https://link.springer.com/journal/44321/submission-guidelines#cms-Revised-submissions. We perform an initial quality control of all revised manuscripts before re-review; failure to include requested items will delay the evaluation of your revision.

We require:

1) A .docx formatted version of the manuscript text (including legends for main figures, EV figures and tables). Please make sure that the changes are highlighted to be clearly visible.

2) Individual production quality figure files as .eps, .tif, .jpg (one file per figure). For guidance, download the 'Figure Guide PDF': https://media.springernature.com/original/springer-cms/rest/v1/content/27825798/data/v1.

3) A .docx formatted letter INCLUDING the reviewers' reports and your detailed point-by-point responses to their comments. As part of the EMBO Press transparent editorial process, the point-by-point response is part of the Review Process File (RPF), which will be published alongside your paper.

4) A complete author checklist, which you can download from our author guidelines. Please insert information in the checklist that is also reflected in the manuscript. The completed author checklist will also be part of the RPF.

5) Please note that all corresponding authors are required to supply an ORCID ID for their name upon submission of a revised manuscript.

6) It is mandatory to include a 'Data Availability' section after the Materials and Methods. Before submitting your revision, primary datasets produced in this study need to be deposited in an appropriate public database, and the accession numbers and database listed under 'Data Availability'. Please remember to provide a reviewer password if the datasets are not yet public.

In case you have no data that requires deposition in a public database, please state so in this section. Note that the Data Availability Section is restricted to new primary data that are part of this study.

7) For data quantification: please specify the name of the statistical test used to generate error bars and P values, the number (n) of independent experiments (specify technical or biological replicates) underlying each data point and the test used to calculate p-values in each figure legend. The figure legends should contain a basic description of n, P and the test applied. Graphs must include a description of the bars and the error bars (s.d., s.e.m.).

8) At EMBO Press we ask authors to provide source data for the main manuscript figures. You will receive a separate email with instructions for providing source data with your revised manuscript, including how to upload and organize the files.

9) Our journal encourages inclusion of *data citations in the reference list* to directly cite datasets that were re-used and obtained from public databases. Data citations in the article text are distinct from normal bibliographical citations and should directly link to the database records from which the data can be accessed. In the main text, data citations are formatted as follows: "Data ref: Smith et al, 2001" or "Data ref: NCBI Sequence Read Archive PRJNA342805, 2017". In the Reference list, data citations must be labeled with "[DATASET]". A data reference must provide the database name, accession number/identifiers and a resolvable link to the landing page from which the data can be accessed at the end of the reference.

10) We replaced Supplementary Information with Expanded View (EV) Figures and Tables that are collapsible/expandable online. A maximum of 5 EV Figures can be typeset. EV Figures should be cited as 'Figure EV1, Figure EV2" etc... in the text and their respective legends should be included in the main text after the legends of regular figures.

- For the figures that you do NOT wish to display as Expanded View figures, they should be bundled together with their legends in a single PDF file called *Appendix*, which should start with a short Table of Content. Appendix figures should be referred to in the main text as: "Appendix Figure S1, Appendix Figure S2" etc.

- Additional Tables/Datasets should be labeled and referred to as Table EV1, Dataset EV1, etc. Legends have to be provided in a separate tab in case of .xls files. Alternatively, the legend can be supplied as a separate text file (README) and zipped together with the Table/Dataset file.

11) The paper explained: EMBO Molecular Medicine articles are accompanied by a summary of the articles to emphasize the major findings in the paper and their medical implications for the non-specialist reader. Please provide a draft summary of your article highlighting

- the medical issue you are addressing,

- the results obtained and

- their clinical impact.

This may be edited to ensure that readers understand the significance and context of the research. Please refer to any of our published articles for an example.

12) Author contributions: You will be asked to provide CRediT (Contributor Role Taxonomy) terms in the submission system. These replace a narrative author contribution section in the manuscript.

13) A Conflict of Interest statement should be provided in the main text.

14) Every published paper includes a 'Synopsis' to further enhance discoverability. Synopses are displayed on the journal webpage and are freely accessible to all readers. They include a short stand first (maximum of 300 characters, including space) as well as 2-5 one-sentences bullet points that summarizes the paper. Please write the bullet points to summarize the key NEW findings. They should be designed to be complementary to the abstract - i.e. not repeat the same text. We encourage inclusion of key acronyms and quantitative information (maximum of 30 words / bullet point). Please use the passive voice. Please attach these in a separate file or send them by email, we will incorporate them accordingly.

Please also suggest a striking image or visual abstract to illustrate your article as a PNG file 550 px wide x 300-600 px high.

15) Include a Reagents and Tools Table as part of the Methods section, which can be downloaded from our author guidelines.

EMBO Molecular Medicine has a "scooping protection" policy, whereby similar findings that are published by others during review or revision are not a criterion for rejection. Should you decide to submit a revised version, I do ask that you get in touch after three months if you have not completed it, to update us on the status.

***** Reviewer's comments *****

Referee #1 (Remarks for Author):

This manuscript describes a strategy for improving persistence of engineered allogeneic regulatory T cells (EngTregs) through a synthetic NKG2A engager. By combining HLA class I/II KO with the anti-NKG2A engager the cells become insensitive to rejection by both T cells and NK cells. The solution is elegant and the models to test the rejection response in whole PBMC and humanized mice are very clever. Benchmarking against the more classical approach based on HLA-E overexpression reveals a more universal effect also in donors with high frequencies of NKG2C.

The authors find that NKG2A is the dominating inhibitory receptor that determine missing self. Other have recently reported that use of a combination of anti-KIR and anti-NKG2A provide a more robust approach for stealth (Hiura et al, ref 26 in this ms). This may be donor dependent as some rely more on NKG2A and others more on KIR, and might only become visible if all KIRs are considered jointly. Is this perhaps contributing to the residual killing of B2M KO + Mona-HL engineered cells in Figure 2D? Given that the same concept with synthetic receptors against NKG2A was first developed by Hiura et al., this discrepancy merits some additional discussion.

In my mind, the major strength of this paper is the clever long-term whole PBMC models set up to study immune evasion. Although it is tempting as a reviewer to ask for more selected investigation of the effect of the edits in cell types, subsets to visualize the effects more clearly, I am convinced by the graphs presented in Figure 3B and C even though the scale shows very low percentages.

For robustness, some data could be extended with higher n to get proper statistics. This applies to Figure 1E, Figure 2C and Figure 2D. It would be nice with more autologous cells as a reference in Figure 3C and to include such cells in Figure 3D.

"The NKG2A.9-based engagers behaved similarly to HLA-E (Fig 2C), suggesting NKG2C engagement may still occur with this NKG2A-selective clone." Is this the case? Does it bind to NKG2C in experiments such as the one shown in Figure 1F?

What is the reason for the altered baseline in Figure 5B and does this affect engraftment? Starting with a higher dose could lead to better persistence?

A minimal set of data should be provide to ensure intact function of stealth engineered EngReg cells against the intended targets. Ideally, the benefit of the stealth edits should be examined in a relevant disease model but understand that this is not trivial.

Referee #2 (Remarks for Author):

In this study the authors present a new modification of engineered Treg cells to generate a clinical product capable of persisting in allogeneic recipients, avoiding rejection mediated by both T and NK cells. They first generate engineered Tregs (EngTregs) by knock-in at the FOXP3 locus in purified CD4+ T cells. Then, such EngTregs are further modified by targeting B2M and CIITA to obtain HLA KO Tregs. After having shown by in vitro assays that HLA KO Tregs can escape alloreactivity of T cells but not of NK cells, they indicate that the NKG2A+ NK cell subset is prominently related to such NK-mediated rejection. Then they analyze different NKG2A engagers and identify the Mona-HL bearing the E56A mutation as the most effective in protecting HLA KO Tregs. Then, they proceed with in vivo experiments in humanized mice confirming the enhanced persistency of HLA KO EngTregs expressing the Mona-HL E56A NKG2A engager. Finally, the authors successfully incorporate such improved NKG2A engager in a clinically applicable Treg product previously generated to reduce the effects of autoimmune T cells in type 1 diabetes.

The study is interesting and scientifically sound. The experiments are well-designed, with appropriate controls and details

effectively described in the supplementary figures. The findings are quite clear, with good novelty.

There are only few minor points to be addressed:

1) Introduction section. A preliminary description of the EngTregs that the authors are going to prepare should be inserted in the Introduction section. As well, it is not immediately clear to what extent the HLAKO Tregs are under investigation (or have been investigated) in different studies. Finally, it may be useful to introduce the description of their GNTI-122 and GNTI-148 Treg products and the idea of modulating HLA-recognition by T and NK cells to improve the potential efficacy of such products.
2) Role of NKG2AnegKIRpos NK cells. Data shown in Figure 1 indicate a positive correlation between the frequency of NKG2A+ cells and the extent of "rejection" of HLAKO Tregs. Although the authors briefly mention a couple of studies supporting the idea that NKG2A+ NK cells could be most effective in killing HLAneg targets, the killing potential of KIRposNKG2Aneg NK cells should not be underestimated, as these latter cells are endowed with important cytolytic activity. This point should be discussed in the Discussion section and possible hypotheses may be proposed suggesting why the frequency of KIR+ cells does not correlate with the rejection of HLAKO Tregs.

Dear Editor:

We are pleased to submit our revised manuscript EMM-2026-23583-T "Synthetic NKG2A engager enables persistence of allogeneic HLA knockout engineered regulatory T cells" to EMBO Molecular Medicine.
We thank the Reviewers for their comments and have revised the manuscript per their requests. Please find our point-by-point responses below in blue font. Modified texts in the revised manuscript are tracked with yellow highlight.

Referee #1 (Remarks for Author):

This manuscript describes a strategy for improving persistence of engineered allogeneic regulatory T cells (EngTregs) through a synthetic NKG2A engager. By combining HLA class I/II KO with the anti-NKG2A engager the cells become insensitive to rejection by both T cells and NK cells. The solution is elegant and the models to test the rejection response in whole PBMC and humanized mice are very clever. Benchmarking against the more classical approach based on HLA-E overexpression reveals a more universal effect also in donors with high frequencies of NKG2C.

The authors find that NKG2A is the dominating inhibitory receptor that determine missing self. Other have recently reported that use of a combination of anti-KIR and anti-NKG2A provide a more robust approach for stealth (Hiura et al, ref 26 in this ms). This may be donor dependent as some rely more on NKG2A and others more on KIR, and might only become visible if all KIRs are considered jointly. Is this perhaps contributing to the residual killing of B2M KO + Mona-HL engineered cells in Figure 2D? Given that the same concept with synthetic receptors against NKG2A was first developed by Hiura et al., this discrepancy merits some additional discussion.
We thank the Reviewer for this comment and recognize importance of inhibitory KIR+ NK cells in missing-self rejection. We agree that it is possible those cells can drive residual rejection responses in the presence of an NKG2A engager, and that suppressing inhibitory KIR+ NK cells would be valuable. We have updated the Discussion to include such content and the important contributions of Hiura et al publication in this regard on lines 315-329.

In my mind, the major strength of this paper is the clever long-term whole PBMC models set up to study immune evasion. Although it is tempting as a reviewer to ask for more selected investigation of the effect of the edits in cell types, subsets to visualize the effects more clearly, I am convinced by the graphs presented in Figure 3B and C even though the scale shows very low percentages.
For robustness, some data could be extended with higher n to get proper statistics. This applies to Figure 1E, Figure 2C and Figure 2D. It would be nice with more autologous cells as a reference in Figure 3C and to include such cells in Figure 3D.
To improve the robustness of the data and accuracy of interpretation, we have made the following modifications to the presented Figures:
Figure 1E – We repeated the experiment to double the sample size and applied appropriate statistical analysis.
Figure 2C – We recognize the Reviewer's critique here and in the next comment regarding this data. Accordingly, we removed language that actively interprets NKG2C relationships and removed statistical

analyses from the figure to avoid implicating associations without further empirical evidence. The updated text (lines 135-137) reflects purely an observation and withdraws any inference on the underlying mechanism.
Figure 2D – We repeated the experiment to double the sample size, with a focus on control and NKG2A engager test articles. Appropriate statistical analysis was also applied and the manuscript text has been updated.
Figure 3C-D – We generated an independent batch of HLA+ EngTregs from a distinct donor and repeated the 7-day co-culture rejection assay using the autologous and five allogeneic responders (new Figure 3E). Consistent with Figure 3C, HLA+ target cells only persist in the presence of autologous responders. We then compiled three independent experiments involving HLA+ targets co-cultured with autologous (n=3) and allogeneic PBMCs (n=18), and plotted the outcomes in a new Figure 3F with statistical analysis. Collectively, these data demonstrate the robustness of the assay system, where HLA+ cells are rejected in allogeneic settings but persist at significantly higher levels with autologous PBMCs.

"The NKG2A.9-based engagers behaved similarly to HLA-E (Fig 2C), suggesting NKG2C engagement may still occur with this NKG2A-selective clone." Is this the case? Does it bind to NKG2C in experiments such as the one shown in Figure 1F?
We appreciate the Reviewer critically questioning the basis of this claim. In the absence of empirical data, we accept the critique from the Reviewer and have remove the language: "…suggesting NKG2C engagement may still occur with this NKG2A-selective clone" from line 137. Our original statement lies in the fact that even for low affinity interactions, which often do not permit detectable binding using soluble proteins, can still lead to biologically significant signaling.

What is the reason for the altered baseline in Figure 5B and does this affect engraftment? Starting with a higher dose could lead to better persistence?
Although the starting LNGFR+ cell number is slightly higher for the NKG2A engager group, the total starting dose is actually lower for these engager expressing cells (Fig 5A – 7.5e6 vs 1e7 for other groups). The LNGFR marker transduction efficiency was higher in the engager-expressing cells. To compensate for the higher LNGFR positivity, we dosed less EngTregs in the engager group versus others. Thus, this infusion product should not have had any advantage in persistence due to higher starting numbers. We apologize for any confusion and have updated the text on lines 210-213 to improve clarity.

A minimal set of data should be provide to ensure intact function of stealth engineered EngReg cells against the intended targets. Ideally, the benefit of the stealth edits should be examined in a relevant disease model but understand that this is not trivial.
To demonstrate the intact function of the NKG2A engager-expressing EngTreg, we compared the suppressive function of donor-matched GNTI-148 (stealth engineered) and GNTI-122 (first-generation, no NKG2A engager or HLA KO) research-grade products. Using a bystander suppression assay, both products suppressed a bystander preproinsulin (PPI)-specific T effector in an antigen-dependent manner, and with similar potency (new Fig 6F). These data demonstrate that incorporation of stealth engineering described here does not impact the function of the EngTreg product. The main text has been updated on lines 259-265 and 487-495 to account for the new data.

Referee #2 (Remarks for Author):

In this study the authors present a new modification of engineered Treg cells to generate a clinical product capable of persisting in allogeneic recipients, avoiding rejection mediated by both T and NK cells. They first generate engineered Tregs (EngTregs) by knock-in at the FOXP3 locus in purified CD4+ T cells. Then, such EngTregs are further modified by targeting B2M and CIITA to obtain HLA KO Tregs. After having shown by in vitro assays that HLA KO Tregs can escape alloreactivity of T cells but not of NK cells, they indicate that the NKG2A+ NK cell subset is prominently related to such NK-mediated rejection. Then they analyze different NKG2A engagers and identify the Mona-HL bearing the E56A mutation as the most effective in protecting HLA KO Tregs. Then, they proceed with in vivo experiments in humanized mice confirming the enhanced persistency of HLA KO EngTregs expressing the Mona-HL E56A NKG2A engager. Finally, the authors successfully incorporate such improved NKG2A engager in a clinically applicable Treg product previously generated to reduce the effects of autoimmune T cells in type 1 diabetes.

The study is interesting and scientifically sound. The experiments are well-designed, with appropriate controls and details effectively described in the supplementary figures. The findings are quite clear, with good novelty.

There are only few minor points to be addressed:

1) Introduction section. A preliminary description of the EngTregs that the authors are going to prepare should be inserted in the Introduction section. As well, it is not immediately clear to what extent the HLAKO Tregs are under investigation (or have been investigated) in different studies. Finally, it may be useful to introduce the description of their GNTI-122 and GNTI-148 Treg products and the idea of modulating HLA-recognition by T and NK cells to improve the potential efficacy of such products.
We appreciate the Reviewer's suggestions for additional context to improve readability of the manuscript. We have made significant amendments across the Introduction section (lines 26-91) to include more information on our EngTreg platform (including brief overviews of GNTI-122 and GNTI-148), a recently published study investigating HLA-matched/KO allogeneic Tregs by McCallion et al, and how immune evasion engineering is essential for efficacy of allogeneic products.

2) Role of NKG2AnegKIRpos NK cells. Data shown in Figure 1 indicate a positive correlation between the frequency of NKG2A+ cells and the extent of "rejection" of HLAKO Tregs. Although the authors briefly mention a couple of studies supporting the idea that NKG2A+ NK cells could be most effective in killing HLAneg targets, the killing potential of KIRposNKG2Aneg NK cells should not be underestimated, as these latter cells are endowed with important cytolytic activity. This point should be discussed in the Discussion section and possible hypotheses may be proposed suggesting why the frequency of KIR+ cells does not correlate with the rejection of HLAKO Tregs.
We agree with the Reviewer that KIR+NK cells possess important cytolytic activity and our observation is worth discussing in the manuscript. We proposed several, non-mutually exclusive hypotheses in the Discussion on lines 330-349 to account for why the observed correlation between B2M KO target killing and inhibitory KIR+ NK cells was weaker than NKG2A+ NK cells. Along with response to Reviewer 1, we also expanded the Discussion on lines 315-329 to emphasize the importance of suppressing inhibitory KIR+ NK cells.

We hope the Reviewers and Editor find our revisions satisfactory in addressing the Reviewers' comments, and consider the revised manuscript suitable for publication in EMBO Molecular Medicine. Thank you for your time and consideration.

Sincerely,

Tingxi (Tim) Guo, PhD
GentiBio, Inc

6th Jul 2026

Dear Dr. Guo,

Thank you for submitting your revised manuscript to EMBO Molecular Medicine. Your manuscript will be ready for acceptance pending the following final amendments:

1) Please consider revising the title to better highlight the main findings, e.g. "A synthetic NKG2A engager enables long-term persistence of HLA-deficient allogeneic regulatory T cells".
2) Abstract: I have gone through your text and revised it (see below). Please review it and amend as you see fit:

Allogeneic cell therapies offer a scalable, off-the-shelf alternative to autologous approaches, but immune rejection, particularly by natural killer (NK) cells following human leukocyte antigen (HLA) deletion, remains a major barrier to durable engraftment. Here, we report an improved synthetic NKG2A engager engineered to selectively inhibit NKG2A^{+} NK cells while avoiding activation of NKG2C^{+} subsets, thereby overcoming a key limitation of the natural ligand HLA-E. Engineered regulatory T cells (EngTregs) lacking HLA and expressing the engager were protected from NK cell-mediated cytotoxicity more effectively than previously reported NK inhibitory strategies. In humanized mouse models, EngTregs persisted for up to 12 weeks, whereas unprotected cells were rapidly rejected. Incorporation of the engager into a clinically compatible dual-AAV EngTregs preserved Treg identity and function while conferring resistance to immune rejection. Together, these findings establish selective NKG2A engager as an effective synthetic immune-evasion strategy and provide a clinically translatable approach to enable durable persistence of off-the-shelf EngTregs therapies.

3) In the main manuscript file, please do the following:
- Please address all comments suggested by our data editors listed below:
o Figure legends:
1. Please note that the exact p values are not provided in the legends of figures 1E, 2D, 3D, F; 4C, 6E, F.
2. Please note that information related to n is missing in the legend of figure 5C.
3. Please note that the error bars are not defined in the legends of figures 2D, EV1 G.
- Please update the order and the headings of the manuscript sections to: Abstract / The Paper Explained / Introduction / Results / Discussion / Methods / Data Availability / Acknowledgements / Disclosure and Competing Interests Statement / References / Figure Legends / Expanded View Figure Legends
- In Methods, provide the antibody dilutions that were used for each antibody.
- In Methods, please add information about ethical approval for experiments involving mice.
- Indicate in legends number and nature of replicates and exact p= values, not a range, along with the statistical test used. To keep the figures "clear" some authors found providing an Appendix table Sx with all exact p-values preferable. You are welcome to do this if you want to.
- Please remove Reagents and Tools Table and upload it as a separate .docx file. More information on how to adhere to this format as well as downloadable templates (.docx) for the Reagents and Tools Table can be found in our author guidelines: https://www.embopress.org/page/journal/17574684/authorguide#structuredmethods
- Please add "Disclosure and competing interests statement". We updated our journal's competing interests policy in January 2022 and request authors to consider both actual and perceived competing interests. Please review the policy https://www.embopress.org/competing-interests and update your competing interests if necessary.
- Author contributions: Please remove it from the manuscript and specify author contributions in our submission system. CRediT has replaced the traditional author contributions section because it offers a systematic machine-readable author contributions format that allows for more effective research assessment. You are encouraged to use the free text boxes beneath each contributing author's name to add specific details on the author's contribution. More information is available in our guide to authors:
https://www.embopress.org/page/journal/17574684/authorguide#authorshipguidelines
4) Funding: Please disclose all sources of funding in our submission system and in "Acknowledgements" in the main manuscript file.
5) The Paper Explained: Please add it to the main manuscript text. The paper explained should be divided in 3 sections "Problem", "Results" and "Impact". Please check any of recently published EMBO Mol Med research articles for reference.
6) Synopsis:
- Synopsis image: Please remove the label "Visual Abstract/Synopsis Image" and upload the image as a high-resolution jpeg/png file exactly 550 px-wide x 300 pixels high.
- Synopsis text: Please upload is as a seprate .doc file.
- Please check your synopsis text and image before submission with your revised manuscript. Please be aware that in the proof stage minor corrections only are allowed (e.g., typos).
7) As part of the EMBO Publications transparent editorial process (see our Editorial at http://embomolmed.embopress.org/content/2/9/329), EMBO Molecular Medicine will publish online a Review Process File (RPF) to accompany accepted manuscripts. This file will be published in conjunction with your paper and will include the anonymous referee reports, your point-by-point response and all pertinent correspondence relating to the manuscript. Let us know if you

want to remove or not any figures from it prior to publication. Please note that the Authors checklist will be published at the end of the RPF.
8) Please provide a point-by-point letter INCLUDING my comments as well as the reviewer's reports and your detailed responses (as Word file).

I look forward to reading a new revised version of your manuscript as soon as possible.

Yours sincerely,

Zeljko Durdevic

Zeljko Durdevic
Senior Editor
EMBO Molecular Medicine

*** Instructions to submit your revised manuscript ***

*** PLEASE NOTE *** As part of the EMBO Publications transparent editorial process initiative (see our Editorial at https://www.embopress.org/doi/pdf/10.1002/emmm.201000094), EMBO Molecular Medicine will publish online a Review Process File to accompany accepted manuscripts.

In the event of acceptance, this file will be published in conjunction with your paper and will include the anonymous referee reports, your point-by-point response and all pertinent correspondence relating to the manuscript. If you do NOT want this file to be published, please inform the editorial office at contact@embomolmed.org.

*****

When preparing your revised manuscript, please refer to our guidelines: https://link.springer.com/journal/44321/submission-guidelines#cms-Revised-submissions. We perform an initial quality control of all revised manuscripts before re-review; failure to include requested items will delay the evaluation of your revision.

We require:

1) A .docx formatted version of the manuscript text (including legends for main figures, EV figures and tables). Please make sure that the changes are highlighted to be clearly visible.

2) Individual production quality figure files as .eps, .tif, .jpg (one file per figure). For guidance, download the 'Figure Guide PDF': https://media.springernature.com/original/springer-cms/rest/v1/content/27825798/data/v1.

3) A .docx formatted letter INCLUDING the reviewers' reports and your detailed point-by-point responses to their comments. As part of the EMBO Press transparent editorial process, the point-by-point response is part of the Review Process File (RPF), which will be published alongside your paper.

4) A complete author checklist, which you can download from our author guidelines. Please insert information in the checklist that is also reflected in the manuscript. The completed author checklist will also be part of the RPF.

5) Please note that all corresponding authors are required to supply an ORCID ID for their name upon submission of a revised manuscript.

6) It is mandatory to include a 'Data Availability' section after the Materials and Methods. Before submitting your revision, primary datasets produced in this study need to be deposited in an appropriate public database, and the accession numbers and database listed under 'Data Availability'. Please remember to provide a reviewer password if the datasets are not yet public.

In case you have no data that requires deposition in a public database, please state so in this section. Note that the Data

Availability Section is restricted to new primary data that are part of this study.

7) For data quantification: please specify the name of the statistical test used to generate error bars and P values, the number (n) of independent experiments (specify technical or biological replicates) underlying each data point and the test used to calculate p-values in each figure legend. The figure legends should contain a basic description of n, P and the test applied. Graphs must include a description of the bars and the error bars (s.d., s.e.m.).

8) At EMBO Press we ask authors to provide source data for the main manuscript figures. You will receive a separate email with instructions for providing source data with your revised manuscript, including how to upload and organize the files.

9) Our journal encourages inclusion of *data citations in the reference list* to directly cite datasets that were re-used and obtained from public databases. Data citations in the article text are distinct from normal bibliographical citations and should directly link to the database records from which the data can be accessed. In the main text, data citations are formatted as follows: "Data ref: Smith et al, 2001" or "Data ref: NCBI Sequence Read Archive PRJNA342805, 2017". In the Reference list, data citations must be labeled with "[DATASET]". A data reference must provide the database name, accession number/identifiers and a resolvable link to the landing page from which the data can be accessed at the end of the reference.

10) We replaced Supplementary Information with Expanded View (EV) Figures and Tables that are collapsible/expandable online. A maximum of 5 EV Figures can be typeset. EV Figures should be cited as 'Figure EV1, Figure EV2" etc... in the text and their respective legends should be included in the main text after the legends of regular figures.

- For the figures that you do NOT wish to display as Expanded View figures, they should be bundled together with their legends in a single PDF file called *Appendix*, which should start with a short Table of Content. Appendix figures should be referred to in the main text as: "Appendix Figure S1, Appendix Figure S2" etc.

- Additional Tables/Datasets should be labeled and referred to as Table EV1, Dataset EV1, etc. Legends have to be provided in a separate tab in case of .xls files. Alternatively, the legend can be supplied as a separate text file (README) and zipped together with the Table/Dataset file.

11) The paper explained: EMBO Molecular Medicine articles are accompanied by a summary of the articles to emphasize the major findings in the paper and their medical implications for the non-specialist reader. Please provide a draft summary of your article highlighting

- the medical issue you are addressing,

- the results obtained and

- their clinical impact.

This may be edited to ensure that readers understand the significance and context of the research. Please refer to any of our published articles for an example.

12) Author contributions: You will be asked to provide CRediT (Contributor Role Taxonomy) terms in the submission system. These replace a narrative author contribution section in the manuscript.

13) A Conflict of Interest statement should be provided in the main text.

14) Every published paper includes a 'Synopsis' to further enhance discoverability. Synopses are displayed on the journal webpage and are freely accessible to all readers. They include a short stand first (maximum of 300 characters, including space) as well as 2-5 one-sentences bullet points that summarizes the paper. Please write the bullet points to summarize the key NEW findings. They should be designed to be complementary to the abstract - i.e. not repeat the same text. We encourage inclusion of key acronyms and quantitative information (maximum of 30 words / bullet point). Please use the passive voice. Please attach these in a separate file or send them by email, we will incorporate them accordingly.

Please also suggest a striking image or visual abstract to illustrate your article as a PNG file 550 px wide x 300 px high.

15) Include a Reagents and Tools Table as part of the Methods section, which can be downloaded from our author guidelines.

EMBO Molecular Medicine has a "scooping protection" policy, whereby similar findings that are published by others during review or revision are not a criterion for rejection. Should you decide to submit a revised version, I do ask that you get in touch after three months if you have not completed it, to update us on the status.

*Additional important information regarding Figures

Each figure should be given in a separate file and should have the following resolution:
Graphs 800-1,200 DPI
Photos 400-800 DPI
Colour (only CMYK) 300-400 DPI"

Figures are not edited by the production team. All lettering should be the same size and style; figure panels should be indicated by capital letters (A, B, C etc). Gridlines are not allowed except for log plots. Figures should be numbered in the order of their appearance in the text with Arabic numerals. Each Figure must have a separate legend and a caption is needed for each panel.

*Additional important information regarding figures and illustrations can be found at
https://media.springernature.com/original/springer-cms/rest/v1/content/27825798/data/v1

***** Reviewer's comments *****

Referee #1 (Comments on Novelty/Model System for Author):

I believe this is a promising approach to avoid NK cell mediated rejection of allogeneic HLA-deficient Tregs

Referee #1 (Remarks for Author):

Authors have addressed all concerns noted in review.

The authors addressed the remaining editorial issues.

3rd Aug 2026

Dear Dr. Guo,

We are pleased to inform you that your manuscript is accepted for publication and is now being sent to our publisher to be included in the next available issue of EMBO Molecular Medicine.

Your manuscript will be processed for publication by EMBO Press. It will be copy edited and you will receive page proofs prior to publication. Please note that you will be contacted by Springer Nature Author Services to complete licensing and payment information.

You may qualify for financial assistance for your publication charges - either via a Springer Nature fully open access agreement or an EMBO initiative. Check your eligibility: https://link.springer.com/journal/44321/how-to-publish-with-us

Should you be planning a Press Release on your article, please notify the production team via this form in order to coordinate publication and release dates: https://forms.cloud.microsoft/e/eH4A8JVP6g

If you have any questions, please do not hesitate to contact the Editorial Office. Thank you for your contribution to EMBO Molecular Medicine.

Yours sincerely,
Zeljko Durdevic

Zeljko Durdevic
Senior Editor
EMBO Molecular Medicine

>>> Please note that it is EMBO Molecular Medicine policy for the transcript of the editorial process (containing referee reports and your response letter) to be published as an online supplement to each paper. If you do NOT want this, you will need to inform the Editorial Office via email immediately. More information is available here: https://link.springer.com/partners/embo-press/editorial-policies#Peer%20review

EMBO Press Author Checklist

Corresponding Author Name: Tingxi Guo	
Journal Submitted to: EMBO Molecular Medicine	
Manuscript Number: EMM-2026-23583-T	

USEFUL LINKS FOR COMPLETING THIS FORM
The EMBO Journal - Author Guidelines
EMBO Reports - Author Guidelines
Molecular Systems Biology - Author Guidelines
EMBO Molecular Medicine - Author Guidelines

Reporting Checklist for Life Science Articles (updated January
This checklist is adapted from Materials Design Analysis Reporting (MDAR) Checklist for Authors. MDAR establishes a minimum set of requirements in transparent reporting in the life sciences (see Statement of Task: 10.31222/osf.io/9sm4x). Please follow the journal's guidelines in preparing your
Please note that a copy of this checklist will be published alongside your article.

Abridged guidelines for figures
1. Data
The data shown in figures should satisfy the following conditions:
- the data were obtained and processed according to the field's best practice and are presented to reflect the results of the experiments in an accurate and unbiased manner.
- ideally, figure panels should include only measurements that are directly comparable to each other and obtained with the same assay.
- plots include clearly labeled error bars for independent experiments and sample sizes. Unless justified, error bars should not be shown for technical
- if n<5, the individual data points from each experiment should be plotted. Any statistical test employed should be justified.
- Source Data should be included to report the data underlying figures according to the guidelines set out in the authorship guidelines on Data

2. Captions
Each figure caption should contain the following information, for each panel where they are relevant:
- a specification of the experimental system investigated (eg cell line, species name).
- the assay(s) and method(s) used to carry out the reported observations and measurements.
- an explicit mention of the biological and chemical entity(ies) that are being measured.
- an explicit mention of the biological and chemical entity(ies) that are altered/varied/perturbed in a controlled manner.
- the exact sample size (n) for each experimental group/condition, given as a number, not a range;
- a description of the sample collection allowing the reader to understand whether the samples represent technical or biological replicates (including how many animals, litters, cultures, etc.).
- a statement of how many times the experiment shown was independently replicated in the laboratory.
- definitions of statistical methods and measures:
  - common tests, such as t-test (please specify whether paired vs. unpaired), simple χ^2 tests, Wilcoxon and Mann-Whitney tests, can be unambiguously identified by name only, but more complex techniques should be described in the methods section;
  - are tests one-sided or two-sided?
  - are there adjustments for multiple comparisons?
  - exact statistical test results, e.g., P values = x but not P values < x;
  - definition of 'center values' as median or average;
  - definition of error bars as s.d. or s.e.m.

Please complete ALL of the questions below.
Select "Not Applicable" only when the requested information is not relevant for your study.

Materials

Newly Created Materials	Information included in the manuscript?	In which section is the information available? (Reagents and Tools Table, Materials and Methods, Figures, Data Availability Section)
New materials and reagents need to be available; do any restrictions apply?	Not Applicable	

Antibodies	Information included in the manuscript?	In which section is the information available? (Reagents and Tools Table, Materials and Methods, Figures, Data Availability Section)
For **antibodies** provide the following information:<br>- Commercial antibodies: RRID (if possible) or supplier name, catalogue number and or/clone number<br>- Non-commercial: RRID or citation	Yes	Reagents and Tools Table

DNA and RNA sequences	Information included in the manuscript?	In which section is the information available? (Reagents and Tools Table, Materials and Methods, Figures, Data Availability Section)
**Short novel DNA or RNA including primers, probes**: provide the sequences.	Not Applicable	

Cell materials	Information included in the manuscript?	In which section is the information available? (Reagents and Tools Table, Materials and Methods, Figures, Data Availability Section)
**Cell lines:** Provide species information, strain. Provide accession number in repository **OR** supplier name, catalog number, clone number, and/**OR** RRID.	Not Applicable	
**Primary cultures:** Provide species, strain, sex of origin, genetic modification status.	Yes	Materials and Methods
Report if the cell lines were recently **authenticated** (e.g., by STR profiling) and tested for mycoplasma contamination.	Not Applicable	

Experimental animals	Information included in the manuscript?	In which section is the information available? (Reagents and Tools Table, Materials and Methods, Figures, Data Availability Section)
**Laboratory animals or Model organisms:** Provide species, strain, sex, age, genetic modification status. Provide accession number in repository **OR** supplier name, catalog number, clone number, **OR** RRID.	Yes	Reagents and Tools Table
**Animal observed in or captured from the field:** Provide species, sex, and age where possible.	Not Applicable	
Please detail **housing and husbandry conditions**.	Yes	Materials and Methods

Plants and microbes	Information included in the manuscript?	In which section is the information available? (Reagents and Tools Table, Materials and Methods, Figures, Data Availability Section)
**Plants:** provide species and strain, ecotype and cultivar where relevant, unique accession number if available, and source (including location for collected wild specimens).	Not Applicable	
**Microbes:** provide species and strain, unique accession number if available, and source.	Not Applicable	

Human research participants	Information included in the manuscript?	In which section is the information available? (Reagents and Tools Table, Materials and Methods, Figures, Data Availability Section)
If collected and within the bounds of privacy constraints report on age, sex and gender or ethnicity for all study participants.	Not Applicable	

Core facilities	Information included in the manuscript?	In which section is the information available? (Reagents and Tools Table, Materials and Methods, Figures, Data Availability Section)
If your work benefited from core facilities, was their service mentioned in the acknowledgments section?	Not Applicable	

Design

Study protocol	Information included in the manuscript?	In which section is the information available? (Reagents and Tools Table, Materials and Methods, Figures, Data Availability Section)
If study protocol has been **pre-registered, provide DOI in the manuscript**. For clinical trials, provide the trial registration number **OR** cite DOI.	Not Applicable	
Report the **clinical trial registration number** (at ClinicalTrials.gov or equivalent), where applicable.	Not Applicable	

Laboratory protocol	Information included in the manuscript?	In which section is the information available? (Reagents and Tools Table, Materials and Methods, Figures, Data Availability Section)
Provide DOI OR other citation details if **external detailed step-by-step protocols** are available.	Yes	Materials and Methods

Experimental study design and statistics	Information included in the manuscript?	In which section is the information available? (Reagents and Tools Table, Materials and Methods, Figures, Data Availability Section)
Include a statement about **sample size** estimate even if no statistical methods were used.	Yes	Materials and Methods
Were any steps taken to minimize the effects of subjective bias when allocating animals/samples to treatment (e.g. **randomization procedure**)? If yes, have they been described?	Yes	Materials and Methods
Include a statement about **blinding** even if no blinding was done.	Yes	Materials and Methods
Describe **inclusion/exclusion criteria** if samples or animals were excluded from the analysis. Were the criteria pre-established?<br><br>If sample or data points were omitted from analysis, report if this was due to attrition or intentional exclusion and provide justification.	Not Applicable	
For every figure, are **statistical tests** justified as appropriate? Do the data meet the assumptions of the tests (e.g., normal distribution)? Describe any methods used to assess it. Is there an estimate of variation within each group of data? Is the variance similar between the groups that are being statistically compared?	Yes	Materials and Methods

Sample definition and in-laboratory replication	Information included in the manuscript?	In which section is the information available? (Reagents and Tools Table, Materials and Methods, Figures, Data Availability Section)
In the figure legends: state number of times the experiment was **replicated** in laboratory.	Yes	Figures
In the figure legends: define whether data describe **technical or biological replicates**.	Yes	Figures

Ethics

Ethics	Information included in the manuscript?	In which section is the information available? (Reagents and Tools Table, Materials and Methods, Figures, Data Availability Section)
Studies involving **human participants**: State details of **authority granting ethics approval** (IRB or equivalent committee(s), provide reference number for approval.	Not Applicable	
Studies involving **human participants**: Include a statement confirming that **informed consent** was obtained from all subjects and that the experiments conformed to the principles set out in the WMA Declaration of Helsinki and the Department of Health and Human Services Belmont Report.	Not Applicable	
Studies involving **human participants: For publication of patient photos**, include a statement confirming that consent to publish was obtained.	Not Applicable	
Studies involving experimental **animals**: State details of **authority granting ethics approval** (IRB or equivalent committee(s), provide reference number for approval. Include a statement of compliance with ethical regulations.	Yes	Materials and Methods
Studies involving **specimen and field samples:** State if relevant **permits** obtained, provide details of authority approving study; if none were required, explain why.	Not Applicable	

Dual Use Research of Concern (DURC)	Information included in the manuscript?	In which section is the information available? (Reagents and Tools Table, Materials and Methods, Figures, Data Availability Section)
Could your study fall under dual use research restrictions? Please check biosecurity documents and list of **select agents and toxins** (CDC): https://www.selectagents.gov/sat/list.htm	Not Applicable	
If you used a select agent, is the security level of the lab appropriate and reported in the manuscript?	Not Applicable	
If a study is subject to dual use research of concern regulations, is the name of the **authority granting approval and reference number** for the regulatory approval provided in the manuscript?	Not Applicable	

Reporting

The MDAR framework recommends adoption of discipline-specific guidelines, established and endorsed through community initiatives. Journals have their own policy about requiring specific guidelines and recommendations to complement MDAR.

Adherence to community standards	Information included in the manuscript?	In which section is the information available? (Reagents and Tools Table, Materials and Methods, Figures, Data Availability Section)
State if relevant guidelines or checklists (e.g., **ICMJE, MIBBI, ARRIVE, PRISMA**) have been followed or provided.	Not Applicable	
For **tumor marker prognostic studies,** we recommend that you follow the **REMARK** reporting guidelines (see link list at top right). See author guidelines, under 'Reporting Guidelines'. Please confirm you have followed these guidelines.	Not Applicable	
For **phase II and III randomized controlled trials**, please refer to the **CONSORT** flow diagram (see link list at top right) and submit the CONSORT checklist (see link list at top right) with your submission. See author guidelines, under 'Reporting Guidelines'. Please confirm you have submitted this list.	Not Applicable	

Data Availability

Data availability	Information included in the manuscript?	In which section is the information available? (Reagents and Tools Table, Materials and Methods, Figures, Data Availability Section)
Have **primary datasets** been deposited according to the journal's guidelines (see 'Data Deposition' section) and the respective accession numbers provided in the Data Availability Section?	Not Applicable	
Were **human clinical and genomic datasets** deposited in a public access-controlled repository in accordance to ethical obligations to the patients and to the applicable consent agreement?	Not Applicable	
Are **computational models** that are central and integral to a study available without restrictions in a machine-readable form? Were the relevant accession numbers or links provided?	Not Applicable	
If publicly available data were reused, provide the respective **data citations in the reference list**.	Not Applicable	
