# Supplementary material for: A synthetic NKG2A engager enables long-term persistence of HLA-deficient allogeneic engineered Tregs
Source: EMBO Mol Med. 2026 Aug 27;18(10):3939–60. doi: 10.1038/s44321-026-00507-4 (PMC13658791; doi:10.1038/s44321-026-00507-4)
Supplement: Supplementary file 2 [file 44321_2026_507_MOESM2_ESM.pdf]

Appendix

Table of Contents

Appendix Figure S1……………………………………………………………………………… 2

Appendix Table S1 ……………………………………………………………………………... 3

Appendix Table S2 ……………………………………………………………………………….. 7

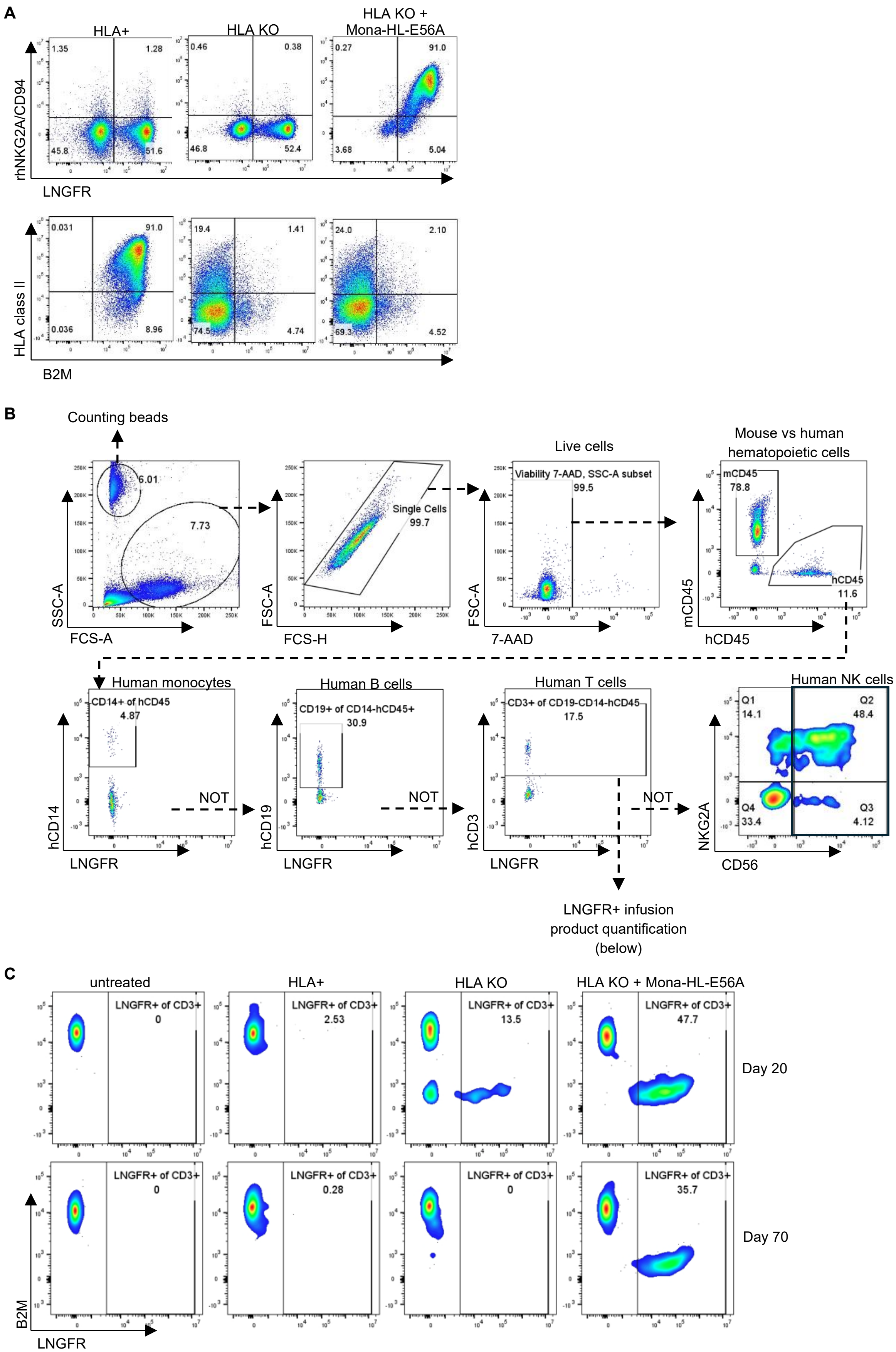

Appendix Figure S1. (A) Representative phenotype of infusion products used in Figures 4 and 5. **(B)** Representative gating strategy for quantifications shown in Figure 5 and Figure EV4. **(C)** Representative flow plots of one animal for quantifying LNGFR+ infusion products in Figure 5 at an early and late timepoint.

Appendix Table S1. Amino acid sequences of NK inhibitors investigated in this study.

NK Inhibitor (Main Figures)	Sequence	Notes
HLA-E	MSRSVALAVLALLSLSGLEAVMAPRTLFLGGGGSGGGGSGGGGSIQRTPKIQVYSRHPAENGKSNFLNCYVSGFHPSDI<br>EVDLLKNGERIEKVEHSDLSFSKDWSFYLLYYTEFTPTEKDEYACRVNHVTLSQPKIVKWDRDMGGGGSGGGGSGGGGS<br>GGGGSGSHSLKYFHTSVSRPGRGEPRFISVGYVDDTQFVRFDNDAASPRMVPRAPWMEQEGSEYWDRETRSARDTAQIF<br>RVNLRTLRGYYNQSEAGSHTLQWMHGCELGPDGRFLRGYEQFAYDGKDYLTLNEDLRSWTAVDTAAQISEQKSNDASEA<br>EHQRAYLEDTCVEWLHKYLEKGKETLLHLEPPKTHVTHHPISDHEATLRCWALGFYPAEITLTWQQDGEGHTQDTELVE<br>TRPAGDGTFQKWAAVVVPSGEEQRYTCHVQHEGLPEPVTLRWKPASQPTIPIVGIIAGLVLLGSVVSGAVVAAVIWRKK<br>SSGGKGGSYSKAEWSDSAQGSESHSL	covalently linked with HLA-G leader peptide and B2M
CD47	MWPLVAALLLGSACCGSAQLLFNKTKSVEFTFCNDTVVIPCFVTNMEAQNTTEVYVKWKFKGRDIYTFDGALNKSTVPT<br>DFSSAKIEVSQLLKGDASLKMDKSDAVSHTGNYTCEVTELTREGETIIELKYRVVSWFSPNENILIVIFPIFAILLFWG<br>QFGIKTLKYRSGGMDEKTIALLVAGLVITVIVIVGAILFVPGEYSLKNATGLGLIVTSTGILILLHYYVFSTAIGLTSF<br>VIAILVIQVIAYILAVVGLSLCIAACIPMHGPLLISGLSILALAQLLGLVYMKFVE	isoform with the shortest intracellular sequence; preferred embodiment in patent US20210292715A1
CD300A TASR	MVLLVTSLLLCELPHPAFLLIPDIQMTQSPSSLSASVGDRVTITCRASQDISNYLNWYQQKPGKAPKLLIYYTSRLHSG<br>VPSRFSGSGSGTDYTLTISNLQPEDFATYFCQQGNTLPWTFGQGTKVEIKGSTSGSGKPGSGEGSTKGQVQLVQSGAEV<br>KKPGASVKVSCKASGYTFTSYWMQWVRQAPGQGLEWIGEIDPSDSYTNYNQKFKGRATLTVDTSTSTTYMELSSLTSED<br>TAVYYCARWGMAYGTSSYWYFDVWGRGTLVTVSSAAATTTPIYIWAPLAGTCGVLLLSLVITLYCNKCFCKHRSCFRRN<br>EASRETNNSLTFGPEEALAEQTVFL	V2 in Zhang et al, 2024, Blood Adv
Z199-HL	MALPVTALLLPLALLLHAARPEVQLVESGGGLVKPGGSLKLSCAASGFTFSSYAMSWVRQSPEKRLEWVAEISSGGSYT<br>YYPDTVTGRFTISRDNAKNTLYLEISSLRSEDTAMYYCTRHGDYPRFFDVWGAGTTVTVSSGSTSGSGKPGSGEGSTKG<br>QIVLTQSPALMSASPGEKVTMTCSASSSVSYIYWYQQKPRSSPKPWIYLTSNLASGVPARFSGSGSGTSYSLTISSMEA<br>EDAATYYCQQWSGNPYTFGGGTKLEIKRTTTPAPRPPTPAPTIASQPLSLRPEACRPAAAVHTRGLDFACDAAVLVLLV<br>IVIISLIVLVVIW	
Z199-LH	MALPVTALLLPLALLLHAARPQIVLTQSPALMSASPGEKVTMTCSASSSVSYIYWYQQKPRSSPKPWIYLTSNLASGVP<br>ARFSGSGSGTSYSLTISSMEAEDAATYYCQQWSGNPYTFGGGTKLEIKRGSTSGSGKPGSGEGSTKGEVQLVESGGGLV<br>KPGGSLKLSCAASGFTFSSYAMSWVRQSPEKRLEWVAEISSGGSYTYYPDTVTGRFTISRDNAKNTLYLEISSLRSEDT<br>AMYYCTRHGDYPRFFDVWGAGTTVTVSSTTTPAPRPPTPAPTIASQPLSLRPEACRPAAAVHTRGLDFACDAAVLVLLV<br>IVIISLIVLVVIW	
Mona-HL	MALPVTALLLPLALLLHAARPQVQLVQSGAEVKKPGASVKVSCKASGYTFTSYWMNWVRQAPGQGLEWMGRIDPYDSET<br>HYAQKLQGRVTMTTDTSTSTAYMELRSLRSDDTAVYYCARGGYDFDVGTLYWFFDVWGQGTTVTVSSGSTSGSGKPGSG<br>EGSTKGDIQMTQSPSSLSASVGDRVTITCRASENIYSYLAWYQQKPGKAPKLLIYNAKTLAEGVPSRFSGSGSGTDFTL<br>TISSLQPEDFATYYCQHHYGTPRTFGGGTKVEIKTTTPAPRPPTPAPTIASQPLSLRPEACRPAAAVHTRGLDFACDAA<br>VLVLLVIVIISLIVLVVIW	
Mona-LH	MALPVTALLLPLALLLHAARPDIQMTQSPSSLSASVGDRVTITCRASENIYSYLAWYQQKPGKAPKLLIYNAKTLAEGV<br>PSRFSGSGSGTDFTLTISSLQPEDFATYYCQHHYGTPRTFGGGTKVEIKGSTSGSGKPGSGEGSTKGQVQLVQSGAEVK<br>KPGASVKVSCKASGYTFTSYWMNWVRQAPGQGLEWMGRIDPYDSETHYAQKLQGRVTMTTDTSTSTAYMELRSLRSDDT<br>AVYYCARGGYDFDVGTLYWFFDVWGQGTTVTVSSTTTPAPRPPTPAPTIASQPLSLRPEACRPAAAVHTRGLDFACDAA<br>VLVLLVIVIISLIVLVVIW	

Z270-HL	MALPVTALLLPLALLLHAARPQVQLQQPGAELVRPGASVKLSCKASGYTFTSYWMNWVKQRPEQGLQWIGRIDPYDSET HYSQKFKDKAILTVDKSSSTAYMRLSSLTSEDSAVYYCARGGYDFDVGTLYWFFDVWGAGTTVTVSGSTSGSGKPGSGE GSTKGDIQMTQSPASLSASVGETVTITCRASENIYSYLAWYQQKQGKSPQFLVYNAKTLAEGVPSRFSGSGSGTQFSLK INSLQPEDFGSYYCQHHYGTPRTFGGGTKLEIKTTTPAPRPPTPAPTIASQPLSLRPEACRPAAAVHTRGLDFACDAAV LVLLVIVIISLIVLVVIW	
Z270-LH	MALPVTALLLPLALLLHAARPDIQMTQSPASLSASVGETVTITCRASENIYSYLAWYQQKQGKSPQFLVYNAKTLAEGV PSRFSGSGSGTQFSLKINSLQPEDFGSYYCQHHYGTPRTFGGGTKLEIKGSTSGSGKPGSGEGSTKGQVQLQQPGAELV RPGASVKLSCKASGYTFTSYWMNWVKQRPEQGLQWIGRIDPYDSETHYSQKFKDKAILTVDKSSSTAYMRLSSLTSEDS AVYYCARGGYDFDVGTLYWFFDVWGAGTTVTVSTTTPAPRPPTPAPTIASQPLSLRPEACRPAAAVHTRGLDFACDAAV LVLLVIVIISLIVLVVIW	
1B2-HL	MALPVTALLLPLALLLHAARPQVQLVESGGGLVKPGGSLRLSCAASGFTFSDYYMSWIRQAPGKGLEWVSYISSSGSTI YYADSVKGRFTISRDNAKNSLYLQMNSLRAEDTAVYYCARGFYMDAWGKGTTVTVSSGSTSGSGKPGSGEGSTKGDIQM TQSPSSLSASVGDRVTITCRASQSISSYLNWYQQKPGKAPKLLIYAASSLQSGVPSRFSGSGSGTDFTLTISSLQPEDF GVYYCQYYGISPPFTFGPGTKVDIKRTTTPAPRPPTPAPTIASQPLSLRPEACRPAAAVHTRGLDFACDAAVLVLLVIV IISLIVLVVIW	
1B2-LH	MALPVTALLLPLALLLHAARPDIQMTQSPSSLSASVGDRVTITCRASQSISSYLNWYQQKPGKAPKLLIYAASSLQSGV PSRFSGSGSGTDFTLTISSLQPEDFGVYYCQYYGISPPFTFGPGTKVDIKRGSTSGSGKPGSGEGSTKGQVQLVESGGG LVKPGGSLRLSCAASGFTFSDYYMSWIRQAPGKGLEWVSYISSSGSTIYYADSVKGRFTISRDNAKNSLYLQMNSLRAE DTAVYYCARGFYMDAWGKGTTVTVSSTTTPAPRPPTPAPTIASQPLSLRPEACRPAAAVHTRGLDFACDAAVLVLLVIV IISLIVLVVIW	
NKG2A9-HL	MALPVTALLLPLALLLHAARPEVQLVESGGGLVKPGGSLRLSCAASGFTFSSHSMNWVRQAPGKGLEWVSAISSSSSYI YYADSVKGRFTISRDNAKNSLYLQMNSLRAEDTAVYYCAREEWGLPFDYWGQGTLVTVSSGSTSGSGKPGSGEGSTKGA IQLTQSPSSLSASVGDRVTITCRASQGISSALAWYQQKPGKAPKLLIYDASSLKSGVPSRFSGSGSGTDFTLTISSLQP EDFATYYCQQFNSYPLTFGQGTRLEIKTTTPAPRPPTPAPTIASQPLSLRPEACRPAAAVHTRGLDFACDAAVLVLLVI VIISLIVLVVIW	
NKG2A9-LH	MALPVTALLLPLALLLHAARPAIQLTQSPSSLSASVGDRVTITCRASQGISSALAWYQQKPGKAPKLLIYDASSLKSGV PSRFSGSGSGTDFTLTISSLQPEDFATYYCQQFNSYPLTFGQGTRLEIKGSTSGSGKPGSGEGSTKGEVQLVESGGGLV KPGGSLRLSCAASGFTFSSHSMNWVRQAPGKGLEWVSAISSSSSYIYYADSVKGRFTISRDNAKNSLYLQMNSLRAEDT AVYYCAREEWGLPFDYWGQGTLVTVSSTTTPAPRPPTPAPTIASQPLSLRPEACRPAAAVHTRGLDFACDAAVLVLLVI VIISLIVLVVIW	
Mona-HL-E56A	MALPVTALLLPLALLLHAARPQVQLVQSGAEVKKPGASVKVSCKASGYTFTSYWMNWVRQAPGQGLEWMGRIDPYDSAT HYAQKLQGRVTMTTDTSTSTAYMELRSLRSDDTAVYYCARGGYDFDVGTLYWFFDVWGQGTTVTVSSGSTSGSGKPGSG EGSTKGDIQMTQSPSSLSASVGDRVTITCRASENIYSYLAWYQQKPGKAPKLLIYNAKTLAEGVPSRFSGSGSGTDFTL TISSLQPEDFATYYCQHHYGTPRTFGGGTKVEIKTTTPAPRPPTPAPTIASQPLSLRPEACRPAAAVHTRGLDFACDAA VLVLLVIVIISLIVLVVIW	E56A mutation is denoted in red
**NK Inhibitor (Fig EV1G)**	**Sequence**	**Notes**
LLT1	MHDSNNVEKDITPSELPANPGCLHSKEHSIKATLIWRLFFLIMFLTIIVCGMVAALSAIRANCHQEPSVCLQAACPESW IGFQRKCFYFSDDTKNWTSSQRFCDSQDADLAQVESFQELNFLLRYKGPSDHWIGLSREQGQPWKWINGTEWTRQFPIL GAGECAYLNDKGASSARHYTERKWICSKSDIHV	

HLA-C0501	MSRSVALAVLALLSLSGLEAIQRTPKIQVYSRHPAENGKSNFLNCYVSGFHPSDIEVDLLKNGERIEKVEHSDLSFSKD<br>WSFYLLYYTEFTPTEKDEYACRVNHVTLSQPKIVKWDRDMGGGGSGGGGSGGGGSGGGGSSHSMRYFYTAVSRPGRGEP<br>RFIAVGYVDDTQFVQFDSDAASPRGEPRAPWVEQEGPEYWDRETQKYKRQAQTDRVNLRKLRGYYNQSEAGSHTLQRMY<br>GCDLGPDGRLLRGYNQFAYDGKDYIALNEDLRSWTAADKAAQITQRKWEAAREAEQRRAYLEGTCVEWLRRYLENGKKT<br>LQRAEHPKTHVTHHPVSDHEATLRCWALGFYPAEITLTWQRDGEDQTQDTELVETRPAGDGTFQKWAAVVVPSGEEQRY<br>TCHVQHEGLPEPLTLRWGPSSQPTIPIVGIVAGLAVLAVLAVLGAVMAVVMCRRKSSGGKGGSCSQAASSNSAQGSDES<br>LIACKA	covalently linked with B2M
CD200	MERLVIRMPFSHLSTYSLVWVMAAVVLCTAQVQVVTQDEREQLYTPASLKCSLQNAQEALIVTWQKKKAVSPENMVTFS<br>ENHGVVIQPAYKDKINITQLGLQNSTITFWNITLEDEGCYMCLFNTFGFGKISGTACLTVYVQPIVSLHYKFSEDHLNI<br>TCSATARPAPMVFWKVPRSGIENSTVTLSHPNGTTSVTSILHIKDPKNQVGKEVICQVLHLGTVTDFKQTVNKGYWFSV<br>PLLLSIVSLVILLVLISILLYWKRH	intracellularly truncated
SERPINB9	METLSNASGTFAIRLLKILCQDNPSHNVFCSPVSISSALAMVLLGAKGNTATQMAQALSLNTEEDIHRAFQSLLTEVNK<br>AGTQYLLRTANRLFGEKTCQFLSTFKESCLQFYHAELKELSFIRAAEESRKHINTWVSKKTEGKIEELLPGSSIDAETR<br>LVLVNAIYFKGKWNEPFDETYTREMPFKINQEEQRPVQMMYQEATFKLAHVGEVRAQLLELPYARKELSLLVLLPDDGV<br>ELSTVEKSLTFEKLTAWTKPDCMKSTEVEVLLPKFKLQEDYDMESVLRHLGIVDAFQQGKADLSAMSAERDLCLSKFVH<br>KSFVEVNEEGTEAAAASSCFVVAECCMESGPRFCADHPFLFFIRHNRANSILFCGRFSSP	
E-cadherin	MGPWSRSLSALLLLLQVSSWLCDWVIPPISCPENEKGPFPKNLVQIKSNKDKEGKVFYSITGQGADTPPVGVFIIERET<br>GWLKVTEPLDRERIATYTLFSHAVSSNGNAVEDPMEILITVTDQNDNKPEFTQEVFKGSVMEGALPGTSVMEVTATDAD<br>DDVNTYNAAIAYTILSQDPELPDKNMFTINRNTGVISVVTTGLDRESFPTYTLVVQAADLQGEGLSTTATAVITVTDTN<br>DNPPIFAQPVEAGLQIPAILGILGGILALLILILLLLLFL	extracellularly truncated Cadherin domains 3-5, which are not required for KLRG1 binding; intracellularly truncated
B7-H3	MLRRRGSPGMGVHVGAALGALWFCLTGALEVQVPEDPVVALVGTDATLCCSFSPEPGFSLAQLNLIWQLTDTKQLVHSF<br>AEGQDQGSAYANRTALFPDLLAQGNASLRLQRVRVADEGSFTCFVSIRDFGSAAVSLQVAAPYSKPSMTLEPNKDLRPG<br>DTVTITCSSYQGYPEAEVFWQDGQGVPLTGNVTTSQMANEQGLFDVHSILRVVLGANGTYSCLVRNPVLQQDAHSSVTI<br>TPQRSPTGAVEVQVPEDPVVALVGTDATLRCSFSPEPGFSLAQLNLIWQLTDTKQLVHSFTEGRDQGSAYANRTALFPD<br>LLAQGNASLRLQRVRVADEGSFTCFVSIRDFGSAAVSLQVAAPYSKPSMTLEPNKDLRPGDTVTITCSSYRGYPEAEVF<br>WQDGQGVPLTGNVTTSQMANEQGLFDVHSVLRVVLGANGTYSCLVRNPVLQQDAHGSVTITGQPMTFPPEALWVTVGLS<br>VCLIALLVALAFVC	intracellularly truncated
HLA-G	MSRSVALAVLALLSLSGLEAIQRTPKIQVYSRHPAENGKSNFLNCYVSGFHPSDIEVDLLKNGERIEKVEHSDLSFSKD<br>WSFYLLYYTEFTPTEKDEYACRVNHVTLSQPKIVKWDRDMGGGGSGGGGSGGGGSGGGGSGSHSMRYFSAAVSRPGRGE<br>PRFIAMGYVDDTQFVRFDSDSACPRMEPRAPWVEQEGPEYWEEETRNTKAHAQTDRMNLQTLRGYYNQSEASSHTLQWM<br>IGCDLGSDGRLLRGYEQYAYDGKDYLALNEDLRSWTAADTAAQISKRKCEAANVAEQRRAYLEGTCVEWLHRYLENGKE<br>MLQRADPPKTHVTHHPVFDYEATLRCWALGFYPAEIILTWQRDGEDQTQDVELVETRPAGDGTFQKWAAVVVPSGEEQR<br>YTCHVQHEGLPEPLMLRWKQSSLPTIPIMGIVAGLVVLAAVVTGAAVAAVLWRKKSSD	covalently linked with B2M
HLA-C1502	MSRSVALAVLALLSLSGLEAIQRTPKIQVYSRHPAENGKSNFLNCYVSGFHPSDIEVDLLKNGERIEKVEHSDLSFSKD<br>WSFYLLYYTEFTPTEKDEYACRVNHVTLSQPKIVKWDRDMGGGGSGGGGSGGGGSGGGGSSHSMRYFYTAVSRPGRGEP<br>HFIAVGYVDDTQFVRFDSDAASPRGEPRAPWVEQEGPEYWDRETQNYKRQAQTDRVNLRKLRGYYNQSEAGSHIIQRMY<br>GCDLGPDGRLLRGHDQLAYDGKDYIALNEDLRSWTAADTAAQITQRKWEAAREAEQLRAYLEGTCVEWLRRYLENGKET<br>LQRAEHPKTHVTHHPVSDHEATLRCWALGFYPAEITLTWQRDGEDQTQDTELVETRPAGDGTFQKWAAVVVPSGEEQRY<br>TCHVQHEGLPEPLTLRWEPSSQPTIPIVGIVAGLAVLAVLAVLGAVMAVVMCRRKSSGGKGGSCSQAASSNSAQGSDES<br>LIACKA	covalently linked with B2M

PD-L1	MRIFAVFIFMTYWHLLNAFTVTVPKDLYVVEYGSNMTIECKFPVEKQLDLAALIVYWEMEDKNIIQFVHGEEDLKVQHS<br>SYRQRARLLKDQLSLGNAALQITDVKLQDAGVYRCMISYGGADYKRITVKVNAPYNKINQRILVVDPVTSEHELTCQAE<br>GYPKAEVIWTSSDHQVLSGKTTTTNSKREEKLFNVTSTLRINTTTNEIFYCTFRRLDPEENHTAELVIPELPLAHPPNE<br>RTHLVILGAILLCLGVALTFIFRL	intracellularly truncated

Appendix Table S2. Exact p values (with 8 significant figures) for statistical analyses in this study.

Dataset	Comparison	Statistical test	P value
Figure 1E, top graph	Targets only, HLA-E+ vs HLA-E-	Two-way ANOVA, Bonferroni's multiple comparisons test	>0.99999999
	3730, HLA-E+ vs HLA-E-		0.00002605
	2859, HLA-E+ vs HLA-E-		0.00000099
Figure 1E, bottom graph	Targets only, HLA-E+ vs HLA-E-		0.51096035
	0350, HLA-E+ vs HLA-E-		<0.00000001
	1226, HLA-E+ vs HLA-E-		0.00000036
Figure 2D, top graph	CTV+, 466 vs No NK		<0.00000001
	CTV+, 2105 vs No NK		<0.00000001
	CTV+, 3244 vs No NK		0.00000001
	CTV+, 3730 vs No NK		<0.00000001
	466, CTV+ vs CFSE+		0.55648655
	2105, CTV+ vs CFSE+		0.39232468
	3244, CTV+ CFSE+		0.36221621
	3730, CTV+ vs CFSE+		0.47224899
	No NK, CTV+ vs CFSE+		0.51947959
Figure 2D, bottom graph	CTV+, 466 vs No NK		<0.00000001
	CTV+, 2105 vs No NK		<0.00000001
	CTV+, 3244 vs No NK		<0.00000001
	CTV+, 3730 vs No NK		<0.00000001
	466, CTV+ vs CFSE+		<0.00000001
	2105, CTV+ vs CFSE+		<0.00000001
	3244, CTV+ CFSE+		<0.00000001
	3730, CTV+ vs CFSE+		0.00000008
	No NK, CTV+ vs CFSE+		0.11737747
Figure 3D	No inhibitor vs HLA-E	one-way ANOVA, Dunn's multiple comparisons test	>0.99999999
	No inhibitor vs CD47		0.63315229
	No inhibitor vs CD300A TASR		0.14548166
	No inhibitor vs Mona-HL		0.10973386
	No inhibitor vs Mona-HL-E56A		0.00195533
Figure 3F	Auto vs allo	unpaired t-test	<0.00000001
Figure 4C	Day 14, %, HLA KO vs HLA KO + Mona-HL-E56A		<0.00000001
	Day 14, counts, HLA KO vs HLA KO + Mona-HL-E56A		0.00184196
Figure 6E	%, HLA KO vs HLA KO + Mona-HL-E56A	paired t-test	0.00018839
	#, HLA KO vs HLA KO + Mona-HL-E56A	Wilcoxon test	0.00048828
Figure 6F	PPI76+, IGRP305+, mock vs GNTI-122	Two-way ANOVA, Bonferroni's multiple comparisons test	0.00000003
	PPI76+, IGRP305+, mock vs GNTI-148		0.00000007
	PPI76+, IGRP305+, GNTI-122 vs GNTI-148		>0.99999999
