# Supplementary material for: A synthetic NKG2A engager enables long-term persistence of HLA-deficient allogeneic engineered Tregs
Source: EMBO Mol Med. 2026 Aug 27;18(10):3939–60. doi: 10.1038/s44321-026-00507-4 (PMC13658791; doi:10.1038/s44321-026-00507-4)
Supplement: Supplementary file 3 [file 44321_2026_507_MOESM9_ESM.pdf]

Expanded View Figures

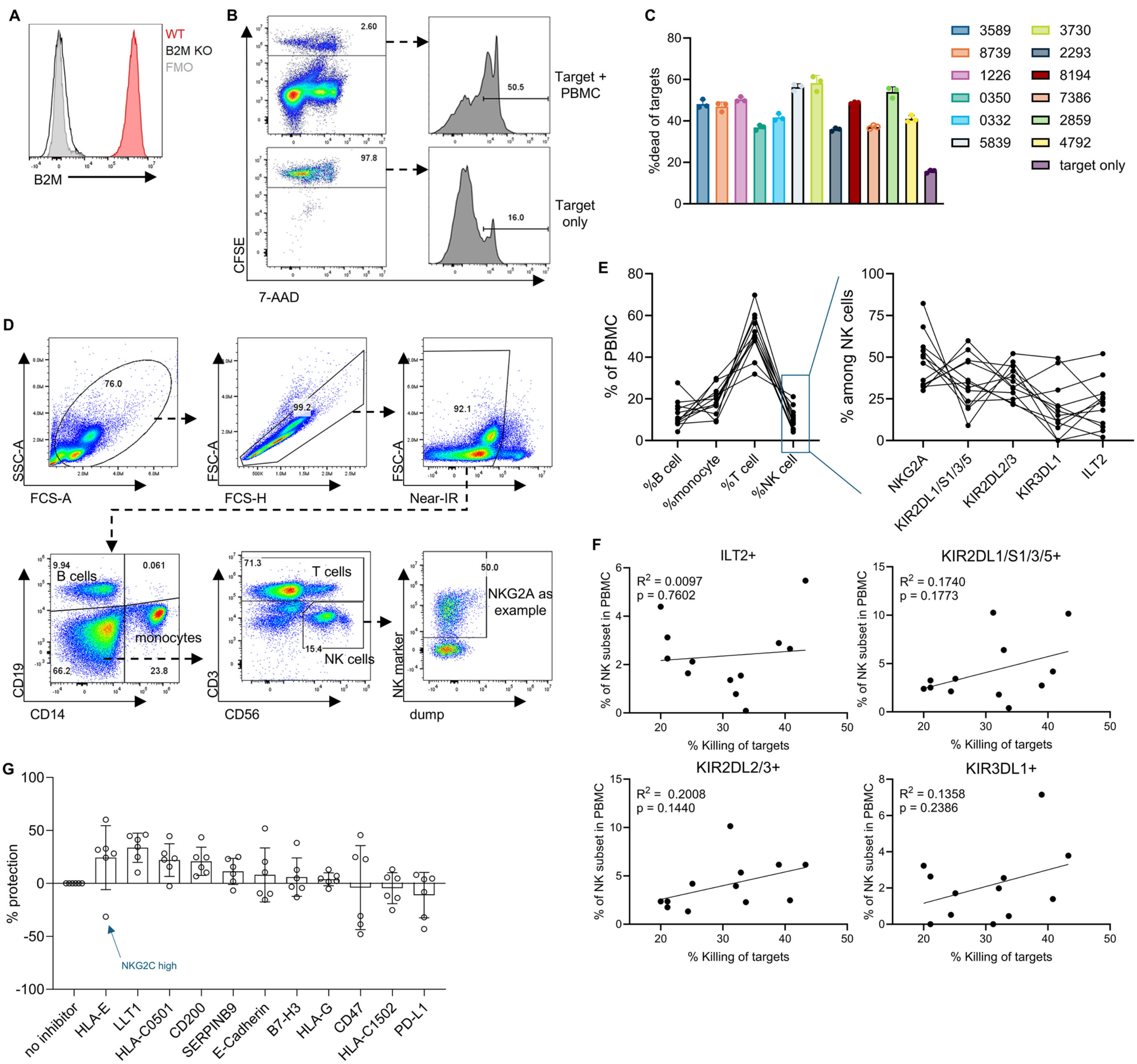

Figure EV1. B2M KO target cell killing and associations with various immune subsets.

(**A**) Surface B2M phenotype of B2M KO EngTregs used in Fig. 1. (**B**) Representative flow plots used for quantifying cell death in CFSE-labeled B2M KO targets with or without PBMCs. Gated on singlets. (**C**) Cell death was quantified as shown in panel (**B**). Error bars denote mean ± SD of technical triplicates. (**D**) Representative gating strategy for quantifying immune subsets in PBMCs. (**E**) Frequencies of indicated immune subsets were quantified in the PBMCs from the 12 donors tested in panel (**C**). Each line denotes one unique donor. (**F**) Linear regression analyses of the percent killing of B2M KO EngTreg targets with the frequency of the indicated NK subset. (**G**) B2M KO Jurkat cells were lentivirally transduced with the indicated transgene and co-cultured with NK cells from six different donors. Percent protection was quantified as described in Methods. Error bars denote mean ± SD.

© The Author(s)

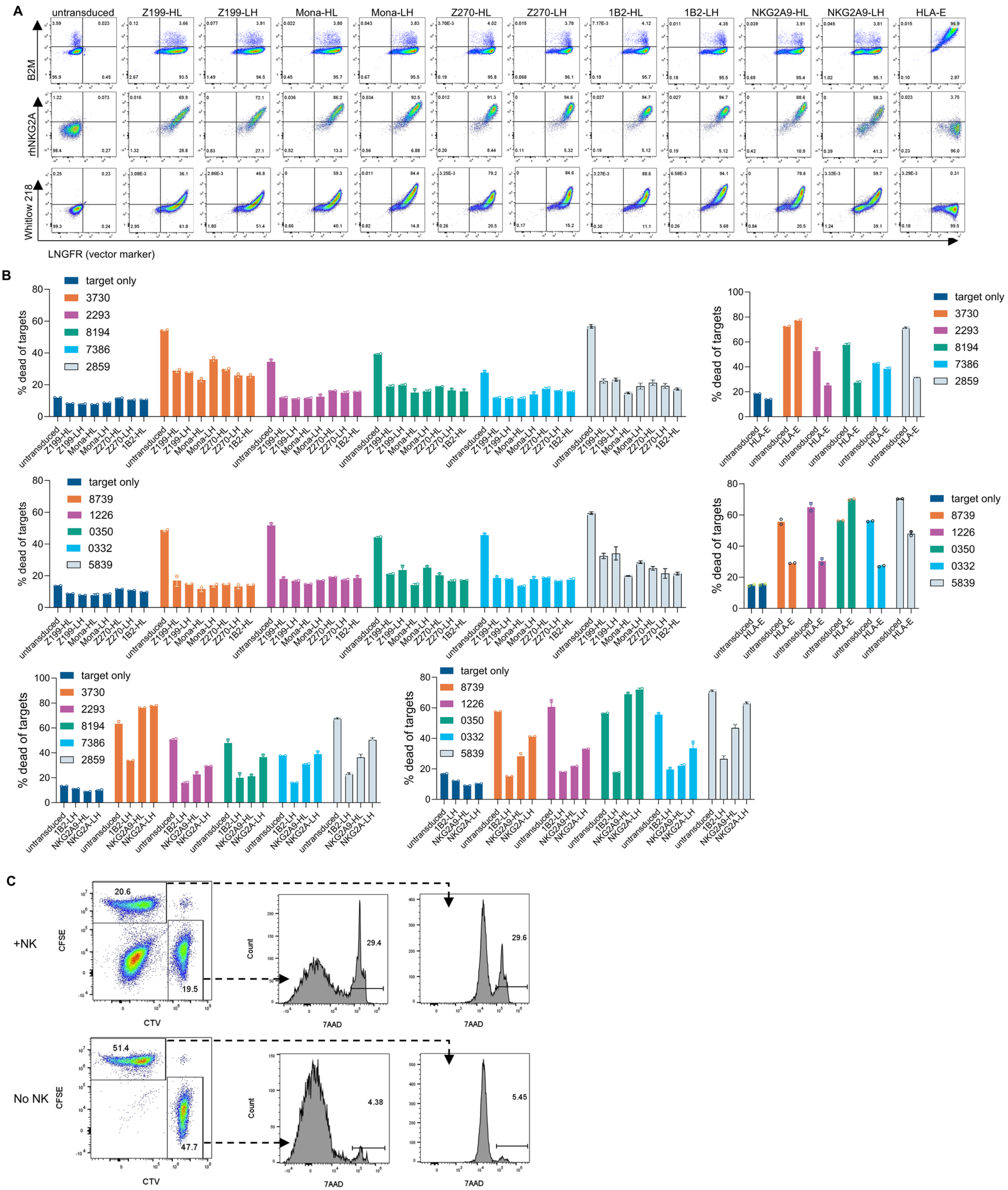
A
untransduced
Z199-HL
Z199-LH
Mona-HL
Mona-LH
Z270-HL
Z270-LH
1B2-HL
1B2-LH
NKG2A9-HL
NKG2A9-LH
HLA-E
B2M
rhNKG2A
Whitlow 218
LNGFR (vector marker)
B
% dead of targets
target only
3730
2293
8194
7386
2859
8739
1226
0350
0332
5839
C
+NK
No NK
CFSE
CTV
Count
7AAD
20.6
19.5
29.4
29.6
51.4
47.7
4.38
5.45

Figure EV2. Expression and protective eff ect of NKG2A engagers.

(**A**) Phenotype of untransduced control and transduced targets, gated on live cells. All lentiviral vectors were marked with truncated LNGFR. NKG2A engagers were detected using recombinant human NKG2A (rhNKG2A) and anti-Whitlow 218 linker antibody. Note that HLA-E is not expected to bind soluble recombinant NKG2A, given the low physiological binding affinity. (**B**) Cell death was quantified and used to calculate percent protection for each condition shown in Fig. 2B. Error bars denote mean ± SD of technical duplicates. Samples assayed on different plates are shown in the respective bar graphs. (**C**) Representative gating strategy for a control LNGFR-transduced EngTreg target used to quantify cell death in Fig. 2D.

© The Author(s)

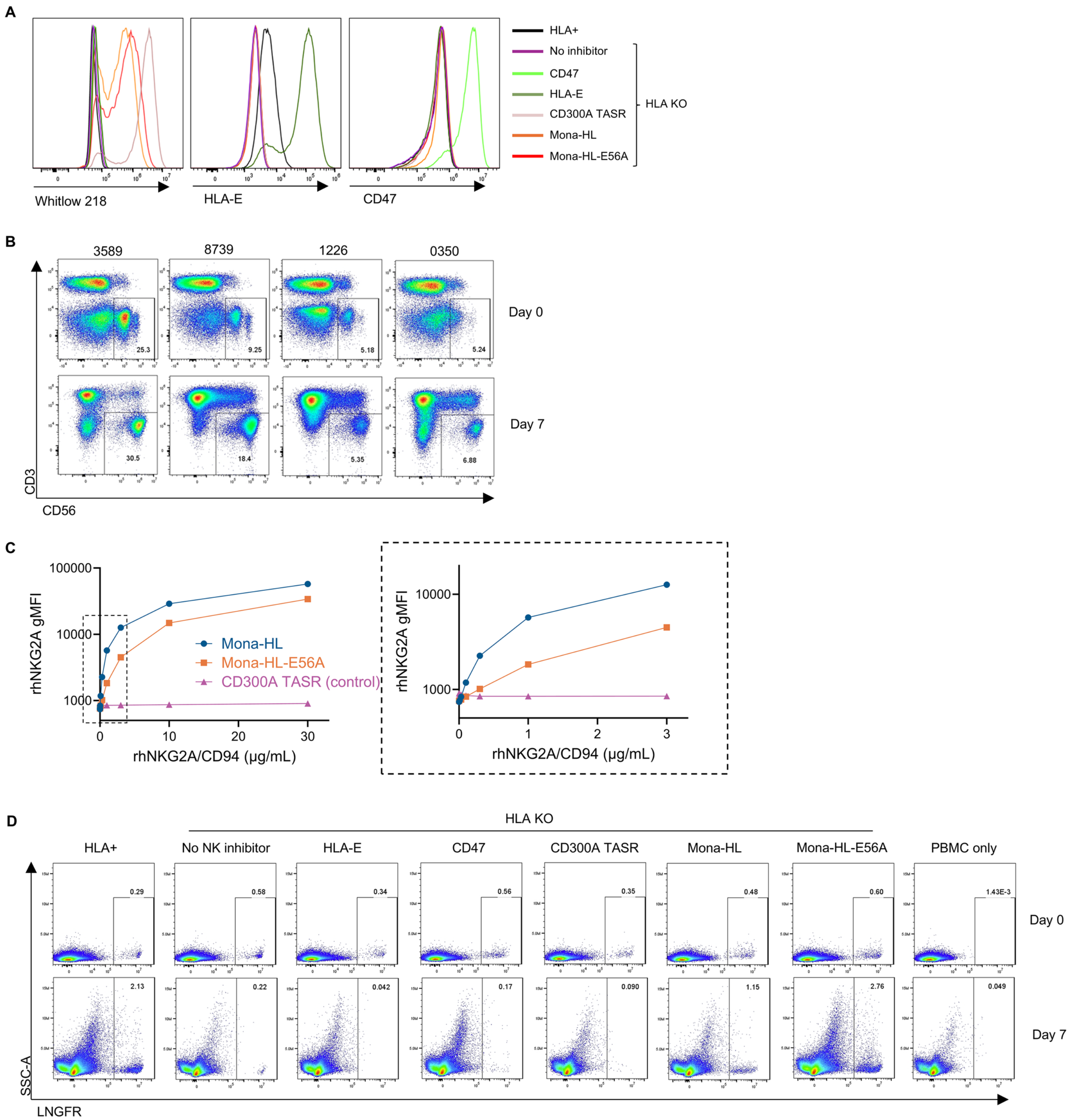

Figure EV3. Expression and function of Mona-HL-E56A engager versus other NK inhibitors.

(**A**) Surface expression of the indicated NK inhibitor on EngTreg test articles used in Fig. 3C. (**B**) Representative flow plots for four PBMC donors co-cultured with HLA KO only target cells demonstrating the presence of CD3-CD56 + NK cells among live, CD14-, LNGFR- cells over the course of the experiment in Fig. 3C. (**C**) Cognate ligand binding strength of Mona-HL and Mona-HL-E56A NKG2A engagers were measured using a cell-based binding assay using titrated concentrations of His-tagged recombinant human NKG2A(rhNKG2A)/CD94, followed by fluorescently labeled anti-His antibody. Fluorescence among LNGFR+ transduced cells were quantified. Cells expressing CD300A TASR were used as a negative control. Error bars denote mean ± SD of technical duplicates or triplicates. The dashed box on the right shows an inset of the low concentration range from the graph on the left. (**D**) Representative flow plots gated on total live CD14- cells for quantifying the percent of LNGFR+ EngTregs shown in Fig. 3C using the PBMC autologous to the EngTregs.

 © The Author(s)

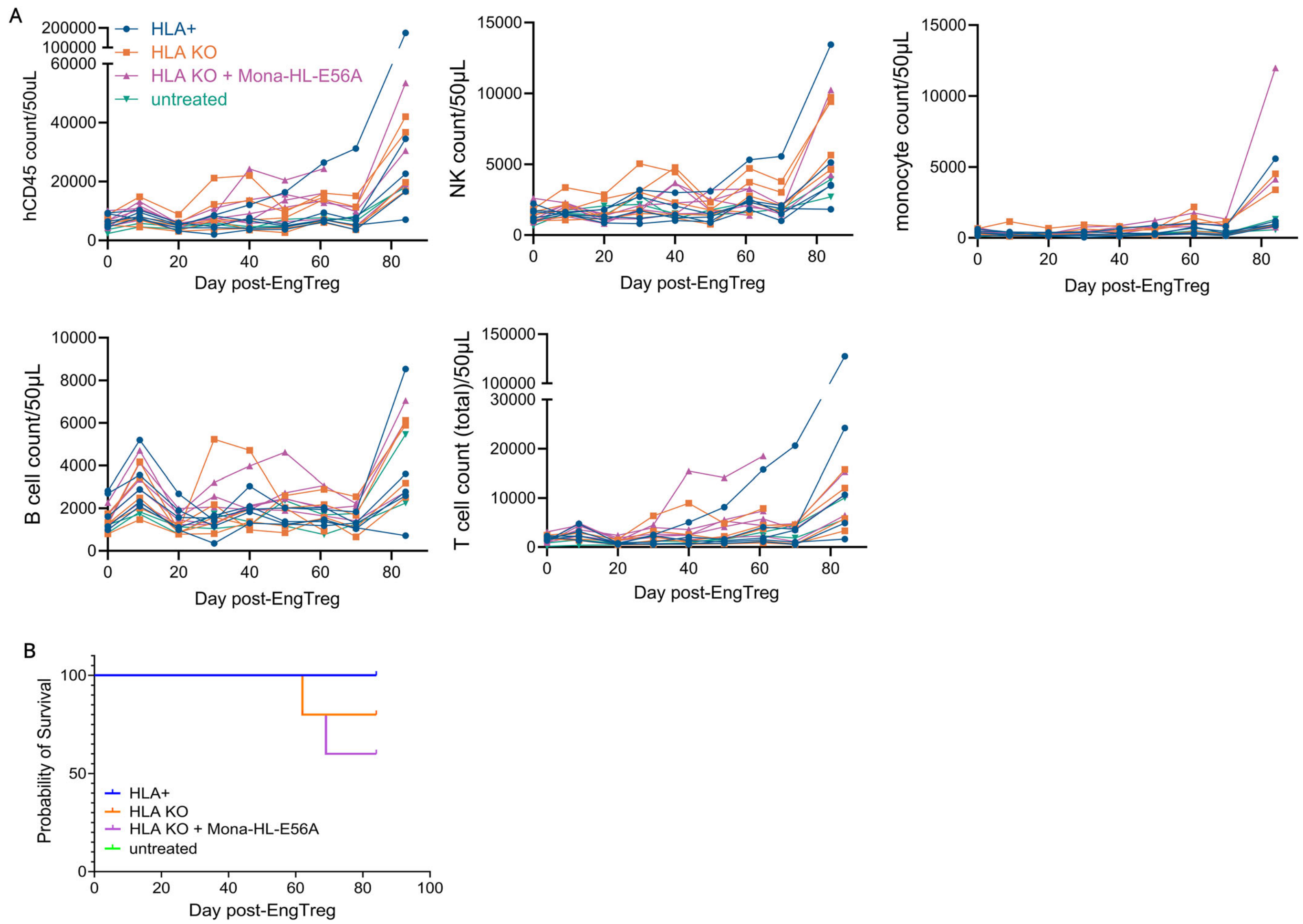

Figure EV4. Peripheral immune subsets and survival over time for HSC-humanized mice.

(**A**) Plots for quantifying the indicated immune population in the peripheral blood of animals in Fig. 5. Each line denotes one unique animal. (**B**) 1 and 2 animals in the HLA KO and HLA KO + Mona-HL-E56A group, respectively, were sacrificed at the indicated times based on Body Condition Score. Sample sizes for each group were the same as described in the legend of Fig. 5.

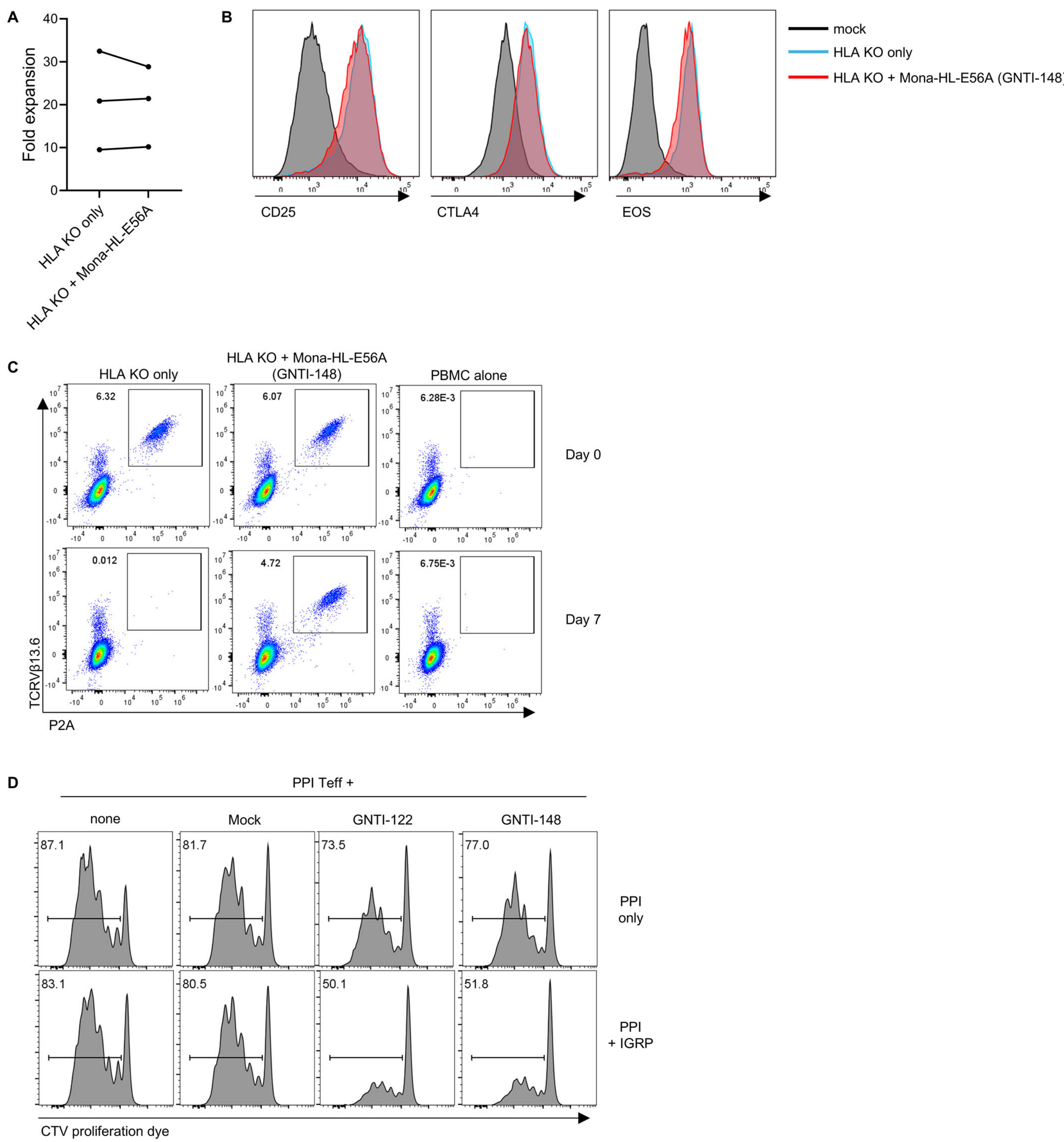

Figure EV5. Phenotype and function of GNTI-148 cells.

(**A**) Fold expansion of research-grade control (HLA KO only) or GNTI-148 (HLA KO + Mona-HL-E56A) EngTregs from day 7 (start of rapamycin) to day 21 (cryopreservation). Each line denotes a unique donor ($n = 3$). (**B**) Representative flow plots gated on live CD4+ cells showing expression of regulatory T cell-associated markers. (**C**) Representative flow plots gated on live CD3+ CD4+ cells for quantifying EngTreg presence in co-cultures shown in Fig. 6D, E. (**D**) Representative flow plots gated on CTV+ CFSE- effector T cells for quantifying % suppression shown in Fig. 6F.

 © The Author(s)
